# Photothermogenetic inhibition of cancer stemness by near-infrared-light-activatable nanocomplexes

**DOI:** 10.1038/s41467-020-17768-3

**Published:** 2020-08-17

**Authors:** Yue Yu, Xi Yang, Sheethal Reghu, Sunil C. Kaul, Renu Wadhwa, Eijiro Miyako

**Affiliations:** 1 Graduate School of Advanced Science and Technology, Japan Advanced Institute of Science and Technology, 1-1 Asahidai, Nomi, Ishikawa 923-1292 Japan; 2 AIST-INDIA DAILAB, DBT-AIST International Center for Translational & Environmental Research (DAICENTER), Cellular and Molecular Biotechnology Research Institute, AIST, Tsukuba, 305-8565 Japan; 3 Present Address: Biomedical Research Institute, National Institute of Advanced Industrial Science & Technology (AIST), Ikeda, 563-8577 Japan

**Keywords:** Biomedical materials, Nanotechnology in cancer

## Abstract

Strategies for eradicating cancer stem cells (CSCs) are urgently required because CSCs are resistant to anticancer drugs and cause treatment failure, relapse and metastasis. Here, we show that photoactive functional nanocarbon complexes exhibit unique characteristics, such as homogeneous particle morphology, high water dispersibility, powerful photothermal conversion, rapid photoresponsivity and excellent photothermal stability. In addition, the present biologically permeable second near-infrared (NIR-II) light-induced nanocomplexes photo-thermally trigger calcium influx into target cells overexpressing the transient receptor potential vanilloid family type 2 (TRPV2). This combination of nanomaterial design and genetic engineering effectively eliminates cancer cells and suppresses stemness of cancer cells in vitro and in vivo. Finally, in molecular analyses of mechanisms, we show that inhibition of cancer stemness involves calcium-mediated dysregulation of the Wnt/β-catenin signalling pathway. The present technological concept may lead to innovative therapies to address the global issue of refractory cancers.

## Abstract (web summary)

Cancer stem cells (CSCs) are known to induce chemotherapy resistance, and cause tumour relapse and metastasis. Here, the authors develop photoactive nanocarbon complexes with second near-infrared photothermal ability to target cancer cells overexpressing the receptor TRPV2 and show it to suppress CSCs through dysregulation of the Wnt/β-catenin signalling pathway.

## Introduction

Chemotherapy is a principal medical remedy for cancer, but resistance to anticancer drugs is a hallmark of malignant tumours that limits the efficacy of cancer treatments. In addition, with disadvantages of cancer selectivity, toxicity and dosage complications of anticancer drugs, the drug design has become less attractive as a long-term solution for cancer. Most anticancer drugs are also limited to natural passive diffusion in the body and are therefore poorly targeted to their sites of action. Moreover, cancer stem cells (CSCs), also known as tumour-initiating cells, are considered responsible for drug resistance and cancer relapse due in large part to their ability to self-renew and promote metastasis^1–3^. There are almost no effective ways to eliminate CSCs except for molecular inhibitors of cancer stemness^4–6^. Yet this still lack enough efficacy, and the design of strategic technologies for inhibiting CSCs remains a major challenge.

Nanomaterials have the potential to control cellular activities with spatial and temporal selectivity through the use of physical treatments, such as magnetic, acoustic and optical excitations^7,8^. However, these approaches have largely been developed independently of the molecular genetics and fail to target the specific cellular activities. Optogenetics employing genetically encoded light-sensitive ion channels or opsins to selectively activate or inhibit neurons could be a possible answer, but its clinical applications are hampered by the limited tissue penetration of visible light^9,10^. Only a few research groups reported strategies using functional nanomaterials for cellular stimulations. Stanley et al. showed that radio wave-induced iron oxide magnetic nanoparticles can regulate transient receptor potential of vanilloid family type 1 (TRPV1) channels, and demonstrated control over insulin activity and cellular glucose levels in mice^11,12^. Gao et al. showed that copper sulfide nanoparticles ameliorate atherosclerosis by photo-thermally modulating TRPV1 signalling activities^13^. Cho et al. achieved remote control of apoptosis by manipulating magnetic nanoparticles with a targeting antibody for death receptor 4 in colon cancer cells^14^. To overcome the challenges of clinical application by regulating cellular activities, we developed biopermeable near-infrared (NIR) light-driven exothermic nanomaterials, including carbon nanotubes (CNTs)^15,16^, carbon nanohorn (CNH)^17–19^, liquid metal nanocomplexes^20^, and semiconducting polymer nanoparticles^21^. In these studies, we demonstrated the use of these nanomaterials for photo-thermally controlling heat-sensitive TRPV1 or TRPV2 ion channels on cell membrane to stimulate cells that we call “photothermogenetics”. We believe that this photothermogenetic approach has the potential to impose gain or loss of function of defined cellular processes by rational molecular design and would be an excellent candidate for developing innovative biomedical applications to the regulation of cancer cell stemness.

In the current study, we develop a photothermogenetics approach using NIR light-activatable CNH complexes, in which tissue-penetrating NIR light is locally converted to thermal energy at levels that are sufficient to stimulate TRPV2 overexpressing cancer cells. NIR light-driven CNHs-mediated photothermogenetics disrupt intracellular Ca^2+^ homoeostasis, suppress oncogenic Wnt/β-catenin signalling through Ca^2+^ dependent degradation of β-catenin, and effectively eliminate cancer cells and inhibit cancer stemness in vitro and in vivo. Our findings contribute to the design of next-generation cancer remedies with various biomedical applications.

## Results

### Preparation and characterisation of CNH complexes

**Fig. 1:**
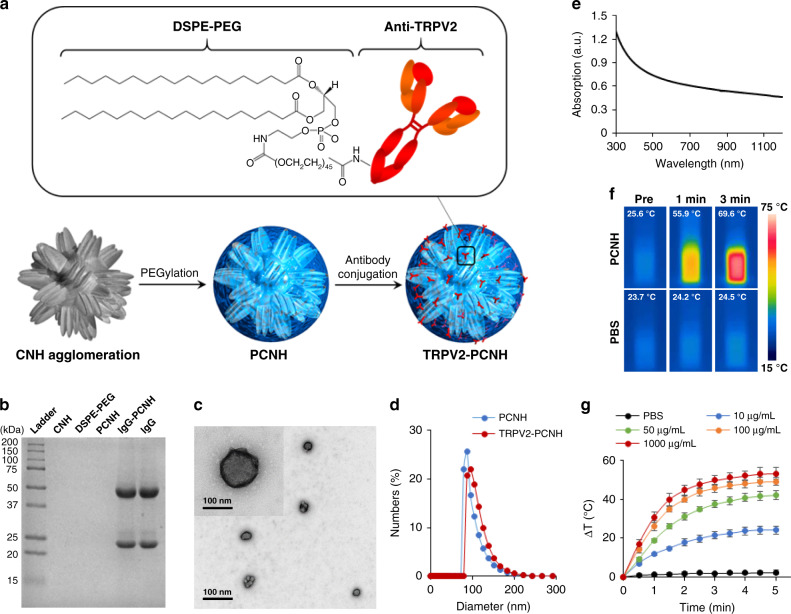
Syntheses and characterisation TRPV2-PCNH. **a** Carbon nanohorn (CNH) agglomerations were self-coated with amphiphilic block phospholipid–PEG copolymers (DSPE–PEG) and TRPV2 antibody was then conjugated to the surface. **b** Sodium dodecyl sulfate-polyacrylamide gele electrophoresis (SDS–PAGE) analyses show successful immobilisation of IgG antibody on PCNH. **c** Representative negative staining transmission electron microscope (TEM) image (a magnified image is shown in the top left corner) and **d** dynamic light scattering (DLS) analyses of TRPV2–PCNH show homogeneous shapes and sizes ranging from 80 to 150 nm. **e** Ultraviolet–visible–near-infrared (UV–Vis–NIR) absorption spectra of TRPV2–PCNH shows a wide absorption range in the NIR region. **f** Thermographic images of phosphate-buffered saline (PBS; 2 ml) and TRPV2–PCNH aqueous dispersions (2 ml, 100 µg ml^−1^) after irradiation with a 1064-nm NIR laser at 1 W (~50 mW mm^−2^). **g** Time-dependent temperature elevation profiles of NIR laser-induced TRPV2–PCNH aqueous dispersions (200 µl) of varying concentrations. Identical volumes of PBS were used as a negative control. Laser power was applied at 1 W (~50 mW mm^−2^). Data are represented as means ± standard deviation (s.d.); *n* = 3 independent experiments.

We selected CNHs as model photo-exothermic nanomaterials due to their strong photothermal conversion effect^17–19^ and high in vitro and in vivo biocompatibility^22–24^. To develop CNHs as photothermogenetic tools for use under physiological conditions, we improved water dispersibility using a PEGylation technique. Because CNHs are hydrophobic, the hydrocarbon chains of phospholipid–PEG (DSPE–PEG) were adsorbed onto the surfaces of CNH agglomerations via van der Waals and hydrophobic interactions, and the hydrophilic PEG chains extend into the aqueous phase to render water solubility. N-Hydroxysuccinimide (NHS) moieties of PEG chain ends were then conjugated with TRPV2 antibody via carbodiimide condensation reactions, therefore enabling the PEGylated CNH (PCNH) to target TRPV2 ion channels selectively (Fig. 1a). TRPV2^25,26^ was chosen as a model target because it can be stimulated at temperatures over 52 °C, at which cancer cell growth is abrogated. TRPV2 receptors also have sufficient heat sensitivity threshold for future clinical applications, because their activation temperature is not reached under conditions of fever or intense physical exercise. Thus, TRPV2-triggered cell activation is controllable by photo-exothermic applications without misregulation. The success of antibody immobilisation was examined using sodium dodecyl sulfate-polyacrylamide gel electrophoresis (SDS–PAGE). After electrophoresis, immunoglobulin (IgG) antibody (isotype control of the TRPV2 antibody)-modified PCNH (IgG–PCNH) clearly showed bands of heavy and light IgG chains (Fig. 1b). Transmission electron microscope (TEM) observations and dynamic light scattering (DLS) analyses also revealed that TRPV2 antibody-modified PCNH (TRPV2–PCNH) contained particles of homogeneous spherical morphology and size, ranging from 80 to 150 nm (Fig. 1c, d). The diameter of TRPV2–PCNH was slightly greater than that of PCNH, further confirming successful conjugation. In addition, the nanoparticles were well dispersed in phosphate-buffered saline (PBS) and remained stable for at least 1 week (Supplementary Fig. 1a).

NIR light has been extensively applied in various sensing, imaging, biological diagnosis, and therapeutic applications, reflecting advantages of remote manipulation, minimal invasion, and high transparency in the range of optical wavelengths for biological tissues^27^. Among NIR regions, the second NIR optical window (NIR-II, 1000–1700 nm) has advantages of deeper tissue penetration and higher maximum permissible light exposure^28^. CNHs have been shown to be capable of absorbing light in NIR-II region^17–19^. We anticipated that the utilisation of NIR-II light-induced CNHs will lead to promising applications in treating deep-tissue-cancer. Indeed, TRPV2–PCNH has high optical absorbance over a wide range of wavelengths, including the NIR-II window (Fig. 1e). Thus, its photothermal properties were studied using a 1064 nm laser. Following irradiation in the NIR range, we envisioned that since nanoparticles transduce electromagnetic radiation into heat, they may therefore be used as in vivo photothermal probes. Thermal images showed that laser irradiation of PCNH dispersions led to remarkable increases in temperature, compared with those with the PBS vehicle control (Fig. 1f). Temperature changes (∆T) of 1-mg ml^−1^ PCNH induced by irradiation at 1 W (~50 mW mm^−2^) were evaluated; these showed increase by ca. 50 °C after 5 min of irradiation (Fig. 1g). Of note, these temperature changes were irradiation time- and PCNH concentration-dependent, offering ease of temperature control. Moreover, photothermal stability was studied by heating and cooling PCNH nanoparticle solutions. The maximum temperatures of PCNH were stable for at least five cycles (Supplementary Fig. 1b). The photothermal conversion efficiency of PCNH at 1064 nm was 59.4%; better than that shown for NIR-II activated competitive gold and polymer nanoparticles^29,30^. These results indicated excellent photothermal properties and suggest potential of our nanocomplexes as activators of thermosensitive ion channels such as TRPV2.

### Targeted photoactivation of TRPV2 by CNHs

**Fig. 2:**
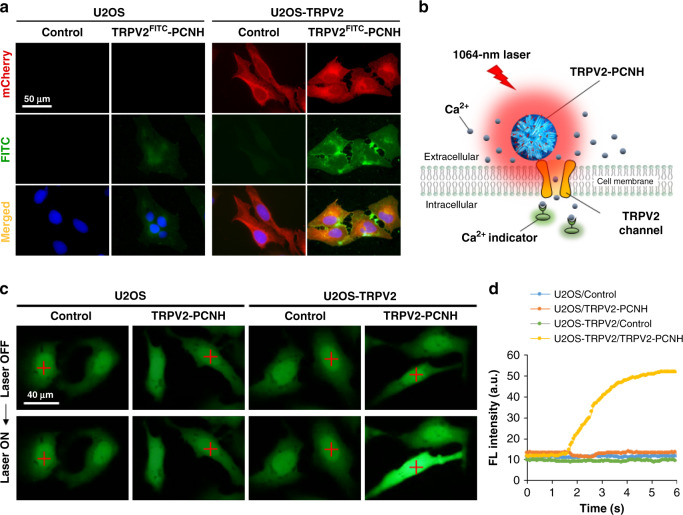
Targeted activation of TRPV2 ion channel by laser-induced TRPV2–PCNH. **a** Fluorescence microscopy imaging of U2OS control and TRPV2-transfected cells incubated with FITC–TRPV2–PCNH (100 µg ml^−1^) for 24 h; TRPV2-PCNH accumulated more efficiently on transfected cells. Nuclei were stained with Hoechst. **b** The schematic illustrates photothermal activation of TRPV2-mediated calcium influx in cancer cells. Internalised Ca^2+^ was monitored in real-time using a Ca^2+^ binding fluorescence indicator. **c** Live cell imaging of U2OS control and nanocomplexes-treated cells shows that TRPV2–PCNH selectively activates Ca^2+^ influx in laser-irradiated TRPV2-transfected cells. Cells were incubated with TRPV2–PCNH (100 µg ml^−1^) for 24 h and were then loaded with the Ca^2+^ indicator (Green) for 30 min before laser stimulation at 0.7 W and ~97 mW mm^−2^ for 1 s. Red crosses indicate irradiated spots. **d** Real-time fluorescence intensities of cells treated with or without TRPV2–PCNH and laser irradiation.

To determine whether TRPV2–PCNH nanoparticles can target cellular TRPV2, we generated mCherry-tagged TRPV2 plasmids and established stably transfected cell lines that overexpress TPRV2 (Supplementary Fig. 2a,b). Since the endogenous TRPV2 expression was nearly absent before transfection (Supplementary Fig. 2b), non-transfected cells were used as negative control for TRPV2 overexpression. Transgenic cells were then incubated with fluorescein isothiocyanate (FITC)-labelled TRPV2–PCNH (TRPV2^FITC^–PCNH). Subsequent fluorescence microscopy analyses showed that U2OS cells overexpressing TRPV2 (U2OS–TRPV2) more efficiently accumulated TRPV2^FITC^–PCNH than non-transfected cells. Green fluorescence of TRPV2^FITC^–PCNH was detected all along the cell membranes and was localised to TRPV2, indicating good surface labelling and targeting (Fig. 2a).

We next attempted to activate TRPV2 ion channels using laser-stimulated TRPV2–PCNH. We expected that the heat from membrane-bound particles would open TRPV2 channels to allow Ca^2+^ influx into the cytosol, and hence monitored the fluorescence of a Ca^2+^ indicator in real-time (Fig. 2b). U2OS cells and their TRPV2 overexpressing counterparts were treated with TRPV2–PCNH nanoparticles for 24 h and were then exposed to 1064-nm laser irradiation for 1 s in fresh medium containing the Ca^2+^ indicator. As shown in Fig. 2c and Supplementary Movies 1 and 2, laser irradiation did not alter the fluorescence intensity of non-transfected U2OS cells, regardless of nanoparticle treatments. In contrast, nanoparticle-treated U2OS–TRPV2 cells showed significant increase in fluorescence intensity upon stimulation (Fig. 2c and Supplementary Movie 4), indicating induction of Ca^2+^ influx. TPRV2-transfected cells without nanoparticle treatments did not show the similar response (Fig. 2c and Supplementary Movie 3), confirming that the observed fluorescence is attributable to photothermal activation of TRPV2. The fact that Ca^2+^ influx occurred within 2 s of laser exposure reflected the favourable photothermal sensitivity of TPRV2–PCNH (Fig. 2d). In further experiments, TRPV2–PCNH efficiently accumulated on TRPV2-transfected C6 glioma cells (Supplementary Fig. 3a) and led to Ca^2+^ influx following NIR laser irradiation. Interestingly, increase in intracellular Ca^2+^ was also propagated from directly irradiated cells to adjacent cells over time, even after switching off the laser (Supplementary Fig. 3b and Supplementary Movie 5). This observation likely reflects cell-to-cell transmission of Ca^2+^ via gap junctions. Induction of Ca^2+^ was also repeatable with successive laser exposures and was reproduced in at least four different views (Supplementary Fig. 4a,b and Supplementary Movie 6). Taken together, these findings clearly demonstrate that laser-induced TRPV2–PCNH remotely controls Ca^2+^ influx into irradiated cells and their neighbours by selectively activating TRPV2 channels.

### Photoinduced apoptosis by CNHs in TRPV2-enriched cells

**Fig. 3:**
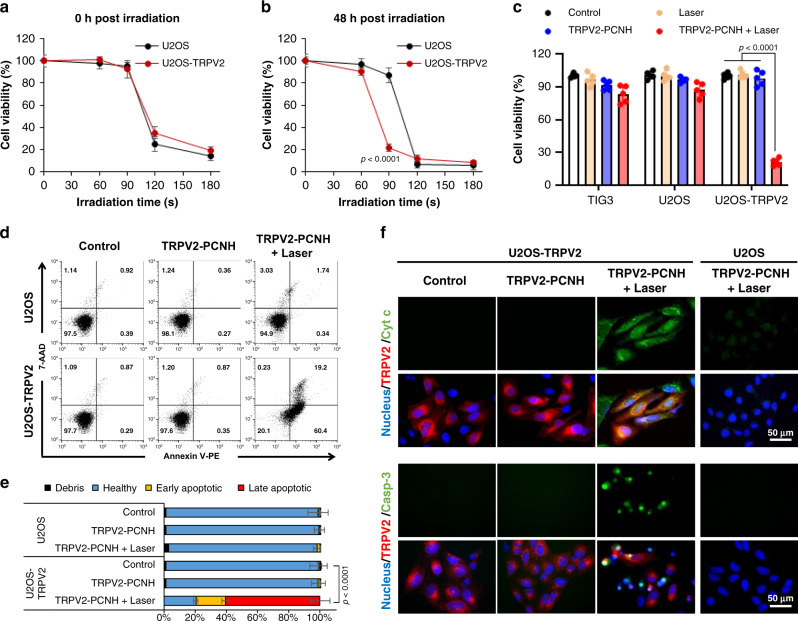
Laser-induced TRPV2–PCNH triggers apoptosis in cancer cells overexpressing TRPV2. **a** Cell viability assays of control and TRPV2-transfected cells were performed immediately after irradiation and show similar responses to increasing irradiation times. Data are represented as means ± standard errors of the mean (s.e.m.); *n* = 3 biologically independent tests. **b** Tests performed at 48 h post irradiation, however, show that U2OS–TRPV2 cells are more sensitive to 90-s pulses of NIR irradiation than control cells. Data are represented as means ± standard errors of the mean (s.e.m.); *n* = 3 biologically independent tests; *P* values were calculated by Student’s two-sided *t* test (comparisons with the 90 s time point in U2OS). **c** Viability of 90-s pulses of laser, TRPV2–PCNH and cotreated cells at 48 h post irradiation; stronger decreases were observed in TRPV2-transfected cells receiving the combination treatment. Data are presented as means ± s.e.m.; *n* = 5 biologically independent tests; *P* values were calculated by Student’s two-sided *t* test. **d** Flow cytometry analysis showing increased apoptotic cell populations in TRPV2-transfected cells after stimulation with TRPV2–PCNH and laser irradiation for 90 s; measurements were performed at 24 h post irradiation. **e** Quantitation from three independent experiments is shown below. Data are expressed as means ± s.e.m. Significant differences in total apoptotic cell numbers were identified using Student’s *t* two-sided test comparisons with control cells; *n* = 3 independent experiments. **f** Fluorescence imaging of control and TRPV2-transfected cells showing increased expression of cytochrome c and caspase-3 in the latter in the following treatments with TRPV2–PCNH and laser irradiation for 90 s; Blue, Hoechest indicates nuclei; Red, mCherry indicates TRPV2; Green, Alexa488 indicates cytochrome c and NucView488 indicates caspase-3. Cells used for all the experiments were treated with TRPV2–PCNH (50 µg ml^−1^) for 24 h before exposure to 1064-nm laser irradiation at 1 W (~50 mW mm^−2^).

Calcium ions are involved in nearly every aspect of cellular processes. To investigate the wider effects of our technology on cell fate determination, we assessed cell viability at 0 and 48 h after laser stimulation of TRPV2–PCNH nanoparticles. As shown in Fig. 3a, b, irradiation time-dependent cytotoxicity was observed. At 0 h post irradiation, U2OS control and TRPV2-transfected cells responded to the treatment in a similar manner (Fig. 3a). However, with additional 48 h incubation, 90-s irradiation pulses caused marked reductions in viability in TRPV2 overexpressing cells, whereas U2OS control cells remained unchanged (Fig. 3b). This delayed response is a common consequence of cell regulatory mechanisms requiring time to determine the cell destinies in the presence of cytotoxic stimuli. Moreover, the higher sensitivity of U2OS–TRPV2 cells to 90-s pulses of irradiation reflects selectivity of TRPV2–PCNH nanoparticles for cells that express TRPV2 (Fig. 3b). To confirm selectivity of TRPV2–PCNH nanoparticles, we performed further experiments using normal TIG3 fibroblast cells. NIR irradiation for 90 s following uptake of 50 µg ml^−1^ of nanoparticles was significantly more cytotoxic to TRPV2 overexpressing cancer cells than normal cells after 48 h (Fig. 3c). Experiments also indicated that TRPV2–PCNH nanoparticles have no intrinsic toxicity (Supplementary Fig. 5). Therefore, we investigated the molecular mechanisms behind these differential effects in TRPV2 control and transfected cells.

Ca^2+^ overload has been shown to instigate apoptosis. Thus, we performed Annexin-V/7-AAD double staining to examine apoptosis in control and nanoparticle-treated cells after laser exposure. As shown in Fig. 3d and Supplementary Fig. 6a, photothermal treatments led to stronger extent of apoptosis in TRPV2-tranfected, as compared to non-transfected, cancer cells. Quantitative data revealed 80% and 60% of apoptotic cells after treatment in U2OS–TRPV2 and C6–TRPV2 cells, respectively (Fig. 3e and Supplementary Fig. 6b). Laser-induced apoptosis in these cells was also confirmed by up-regulation of cytochrome c and caspase 3 (Fig. 3f and Supplementary Fig. 6c). Nanoparticle treatments without laser irradiation again had no effects in these experiments. These results suggest that laser-irradiated TRPV2–PCNH nanoparticles selectively induce Ca^2+^-dependent apoptosis in cells overexpressing TRPV2.

### In vitro attenuation of cancer stemness

**Fig. 4:**
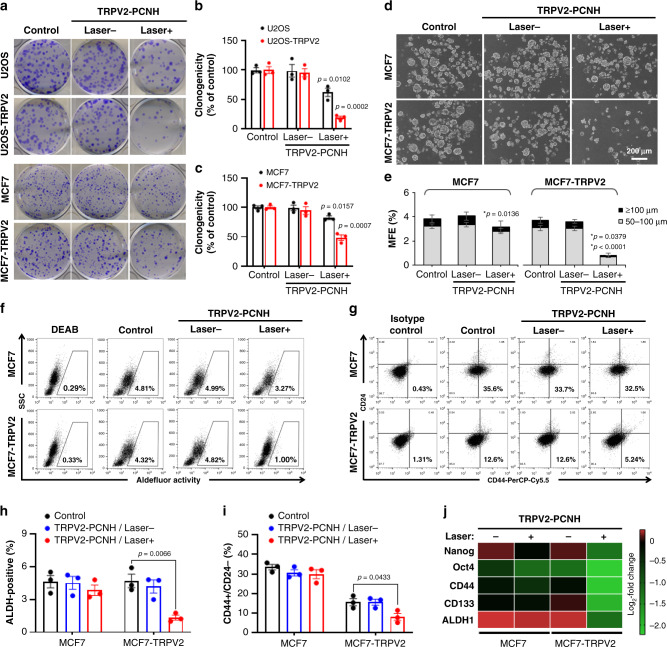
Laser-induced TRPV2–PCNH attenuates stemness in cancer cells overexpressing TRPV2. **a** Colony-forming assays of control and TRPV2-transfected cells show stronger responses of the latter to TRPV2-CNH treatments after laser irradiation. Quantitative clonogenicity assays are shown in **b**, **c**. Data are expressed as means ± s.e.m.; *n* = 3 independent experiments; and differences were identified using Student’s two-sided *t* test comparisons with control cells. **d** Representative mammosphere formation assays of TRPV2-CNH-treated control and TRPV2 overexpressing cells after laser stimulation. **e** Numbers of mammospheres of 50–100 µm and over 100 µm show stronger reductions in mammosphere forming efficiency in MCF7 transfected cells than in parental cells. Data are presented as means ± s.e.m. (*n* = 3 independent experiments). Comparisons with controls were made using Student’s two-sided *t* test; *P: mammosphere 50–100-µm; #P: mammosphere ≥100-µm. **f** Flow cytometry analysis show that laser irradiation decreases ALDH activities and **g** CD44+/CD24− subpopulations in TPRV2-PCNH treated cells overexpressing TRPV2. Cells were treated with diethylaminobenzaldehyde (DEAB) or were stained with isotype antibodies for use as negative controls. Data in **h**, **i** are presented as means ± s.e.m. (*n* = 3 independent experiments). *P* values were determined using Student’s two-sided *t* test. **j** RT-qPCR analyses are represented by a Log2-fold change heatmap. TRPV2-overexpressing cells treated with TRPV2–PCNH and laser irradiation showed stronger declines in the expression of stemness-related markers genes. All cells were treated with TRPV2–PCNH (50 µg ml^−1^) for 24 h, followed by 90 s of laser irradiation at 1 W (~50 mW mm^−2^) and measurements were performed at 24 h post irradiation.

Given that calcium signalling is implicated in cancer cell stemness, which is central to the initiation and progression of malignancies^31^, we determined whether laser-induced TRPV2–PCNH nanoparticles inhibit stem cell properties using colony-forming assays in U2OS and MCF7 cells with or without TRPV2 overexpression. Compared with their untransfected counterparts, TRPV2-overexpressing cells showed greater reductions in clonogenicity after laser irradiation (Fig. 4a), with decreases of 18.7% and 38.5%, respectively (Fig. 4b, c). To further demonstrate in vitro tumorigenic capacity, we performed mammosphere formation assays in stemness-high MCF7 and TRPV2 overexpressing cells. Representative micrographs of mammospheres that were formed after laser treatments show that TRPV2–PCNH-mediated phototherapy potently diminishes numbers and sizes of mammospheres derived from cells overexpressing TRPV2 (Fig. 4d). Quantitative analyses also showed that nanoparticle and laser stimulation strongly prevented mammosphere formation (3.72% to 0.7%) in TRPV2-transfected cells, but only slightly decreased (3.8% to 3.2%) this process in parent MCF7 cells (Fig. 4e). Moreover, being different from MCF7 cells showing comparable sphere-forming frequency before and after treatments (1/22 and 1/30) (Supplementary Fig. 7a), nanoparticle/laser-treated MCF7-TRPV2 cells showed a clear reduction in frequency of cells possessing self-renewal ability (1/29 to 1/105) (Supplementary Fig. 7b). These data would suggest laser-driven TRPV2-PCNH could inhibit tumour regenerative potential and the self-renewal capacity.

In order to investigate whether this technique can enhance chemosensitivity to anticancer drugs, A549 control and TRPV2-transfected cells were pretreated with nanocomplexes combining laser irradiation, followed by 48 h exposure to paclitaxel (PTX). Validation with a range of tested doses to generate dose response curves showed that nanoparticle-mediated photo-stimulation sensitized A549-TRPV2 cells to PTX, of which IC50 value decreased to 2.23 nM (from 9.11 nM) after treatment (Supplementary Fig. 8). In contrast, difference in A549 control cells was modest (5.03 nM to 4.45 nM) (Supplementary Fig. 8). In further analyses of cancer cell stemness, we examined aldehyde dehydrogenase (ALDH) activity using flow cytometry at 24 h post NIR irradiation. Significant reductions in proportions of ALDH-positive cells were observed among MCF7–TRPV2 cells, but not among MCF7 cells (Fig. 4f, h). Accordingly, we observed decreased numbers of CD44^high^/CD24^low^ cells among laser-irradiated MCF7–TRPV2 cells, although TRPV2 transfection somehow reduced CD44^high^ /CD24^low^ subpopulations irrespective of irradiation (Fig. 4g, i). Mean fluorescence intensity of CD44 in MCF7-TRPV2 cells was also decreased after laser stimulation (Supplementary Fig. 9). These data were validated by real-time-quantitative polymerase chain reaction (RT-qPCR) analyses, which confirmed stronger declines in transcriptional expression levels of stemness-related markers in TRPV2 overexpressing MCF7 cells (Fig. 4j). These results collectively demonstrated that TRPV2–PCNH mediated photoactivation of Ca^2+^ influx represses CSC characteristics in TRPV2 overexpressing cells.

### In vivo inhibition of tumour growth by laser-driven CNHs

The data presented above warranted in vivo assessment of the therapeutic efficacy of TRPV2–PCNH nanoparticles using a subcutaneous xenograft nude mouse model. To investigate biological distributions of nanoparticles and quantify TRPV2 targeting effects in vivo, indocyanine green (ICG)-encapsulated PCHN or TRPV2–PCNH nanocomplexes were intravenously injected into mice bearing two tumours on opposite flanks, which were derived from HT-29 cells and TRPV2 derivative cells, respectively. Whole-body fluorescence live imaging showed that ICG-labelled TRPV2–PCNH nanocomplexes accumulated at tumour xenografts, with peak accumulations at 24 h after injection (Supplementary Fig. 10a). Fluorescent signals were stronger in HT-29–TRPV2 tumours than in HT-29 tumours throughout 48-h observations, suggesting sustained targeting of TRPV2–PCNH nanoparticles to TRPV2 overexpressing tumours (Supplementary Fig. 10b). Furthermore, after excising tumours and other major organs at 24 h post injection (Supplementary Fig. 10c), TRPV2 antibody-functionalised nanoparticles (TRPV2–PCNH) were mainly accumulated in tumours overexpressing TRPV2 (HT-29–TRPV2), with more than two-fold higher radiant efficiency than in HT-29 control tumours and vital organs such as liver, lung, spleen, heart and kidney (Supplementary Fig. 10d). In contrast, mice injected with PCNH showed comparable accumulation in the two tumours as in other organs (Supplementary Fig. 10d). These observations further confirm tumour-selective accumulation of TRPV2-targeting nanoparticles.

The pharmacokinetics profile of the Cy5-labelled CNHs was also assessed by fluorometry to determine the concentrations in blood and organs at different time intervals post-injection. Higher uptake was observed in the liver and kidney while no significant uptake was detected in other organs (Supplementary Fig. 11). This is consistent with above fluorescence imaging results. With dominate accumulation in the liver at 1 h post-injection, the nanocomplex concentrations gradually decreased at later time points (Supplementary Fig. 11), suggesting the body clearance. After 14 days, the residual CNHs were decreased to 8.5 % ID g^−1^ and 2.9 % ID g^−1^ in the liver and kidney, respectively (Supplementary Fig. 11), indicating that vast majority was cleared out from the body. In fact, it is well known that CNH has low toxicity and fully biologically degradable, as demonstrated by various systemic biocompatibility analyses^32–34^. From these results, we anticipate that CNHs would be gradually eliminated from biological body over time without any severe side effect.

**Fig. 5:**
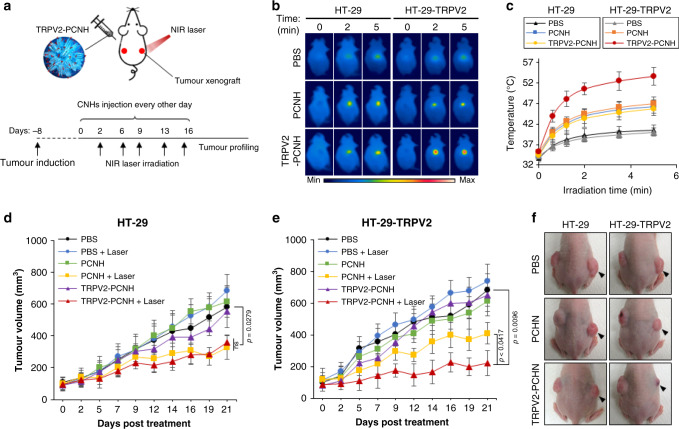
Laser-induced TRPV2-PCNH inhibits tumour progression in in vivo xenograft models with TRPV2 overexpression. **a** In vivo experimental scheme: on day -8, HT-29 control and stably transfected TRPV2 cells were injected into mice to establish tumour xenograft models. On day 0, PBS, PCHN, or TRPV2–PCNH were administered (5 mg kg^−1^) intraperitoneally (i.p.) every other day until day 16. NIR treatments were applied to tumours on the right sides at days 2, 6, 9, 13 and 16 using a 1064-nm laser at 1 W (~50 mW mm^−2^) for 5 min. **b** Thermographic images and **c** laser-induced temperature increases in mice bearing HT-29 or HT-29–TRPV2 tumours. Measurements were performed at 24 h after injection of nanocomplexes. Data are represented as means ± s.d.; *n* = 6 biologically independent mice. **d** HT-29 and **e** HT-29–TRPV2 tumour volumes in different groups of mice during the course of treatment (mean ± s.e.m., *n* = 6 biologically independent mice, two-way ANOVA test). Laser-irradiated subcutaneous xenografts of HT-29–TRPV2 cells in nude mice treated with TRPV2–PCNH showed the greatest reductions in tumour volumes over time. **f** Photographs of HT-29 and HT-29–TRPV2 tumour-bearing mice on day 16; black arrows indicate tumours that were irradiated by laser.

To evaluate the effects of anticancer phototherapy, we established tumour xenograft mouse models using subcutaneous injections with either HT-29 cells or their TRPV2-overexpressing derivatives into the flanks of mice, as illustrated in Fig. 5a. Mice bearing separate tumours from both cell lines were randomly assigned to (1) PBS (blank control); (2) PBS + laser (laser control); (3) PCNH (non-targeted nanoparticle control); (4) PCNH + laser (non-targeted phototherapy control); (5) TRPV2–PCNH (targeted nanoparticle control) and (6) TRPV2–PCNH + laser (targeted phototherapy) groups. Mice were then intraperitoneally administered with nanoparticles at equivalent doses (5 mg kg^−1^) every other day. In NIR irradiation groups, tumours on the right sides were exposed to NIR light (1 W, ~50 mW mm^−2^) for 5 min at fixed time points (2, 6, 9, 13 and 16 days) from 24 h after injections (Fig. 5a). Body surface temperatures were monitored during laser irradiation using a thermographic infrared camera (Fig. 5b). As shown in Fig. 5c, temperatures above 52 °C (TRPV2 activation threshold) occurred only in HT-29–TRPV2 tumours of the TRPV2–PCNH + laser group.

To demonstrate anti-tumour efficacy, we recorded tumour volumes for about 21 days after initiating the therapeutic regimen. In both xenograft tumours, NIR irradiation and nanoparticle treatments did not affect tumour growth when administered alone. Yet in combination, PCNH or TRRV2–PCNH nanoparticle treatments with laser irradiation delayed both HT-29 and HT-29-TRPV2 tumour growth compared with that in mice treated with PBS (Fig. 5d, e). HT-29–TRPV2 tumours were more strongly suppressed under these conditions in the TRRV2–PCNH + Laser group than in the other treatment groups (Fig. 5e), perhaps indicating selective targeting of TRPV2-overexpressing cells by TRRV2–PCNH. HT-29 tumour growth was also suppressed in the TRRV2–PCNH + Laser group compared with the PBS control group, but the effect was comparable to that in the PCNH + Laser group (Fig. 5d). Tumour inhibition by laser-induced TRRV2–PCNH was clearly observed in HT-29–TRPV2 xenografts at day 16 after nanoparticle injections. Moreover, laser-irradiated HT-29–TRPV2 tumours were much smaller than non-irradiated tumours on the opposite flanks of the same mice without irradiation, whereas no irradiation-related differences were observed between HT-29 xenografts (Fig. 5f). Hence, we conclude that TRRV2–PCNH selectively impedes TRPV2 positive tumour progression following NIR laser irradiation in vivo. No significant body weight losses were identified in the mice of any treatment groups, demonstrating that TRRV2–PCNH has low systemic toxicity (Supplementary Fig. 12).

In order to further demonstrate the practicability of the TRPV2-PCNH mediated phototherapy towards future medical use, the anti-tumour efficacy was next evaluated using immunocompetent mice. TRPV2 stable transfected Colon-26 cells was established to develop tumour syngeneic models (Supplementary Fig. 13a). As expected, mice treated with nanocomplexes and laser irradiation showed best performance on tumour growth inhibition (Supplementary Fig. 13b,c). Compared with Colon-26 derived tumours, the size of TRPV2-overexpressing tumours (Colon-26-TRPV2) was reduced in a greater extent (Supplementary Fig. 13b–d). Gradually increased mice body weight in all groups over treatment period confirmed biosafety of this therapy (Supplementary Fig. 13e). Importantly, such cancer therapeutic efficacy was also observed in A549 xenografts with in situ transfection of TRPV2 (Supplementary Fig. 14), indicating this technique is able to work across multiple tumour types without the limitation of endogenous TRPV2 expression.

### In vivo regulation of cancer stemness by laser-driven CNHs

**Fig. 6:**
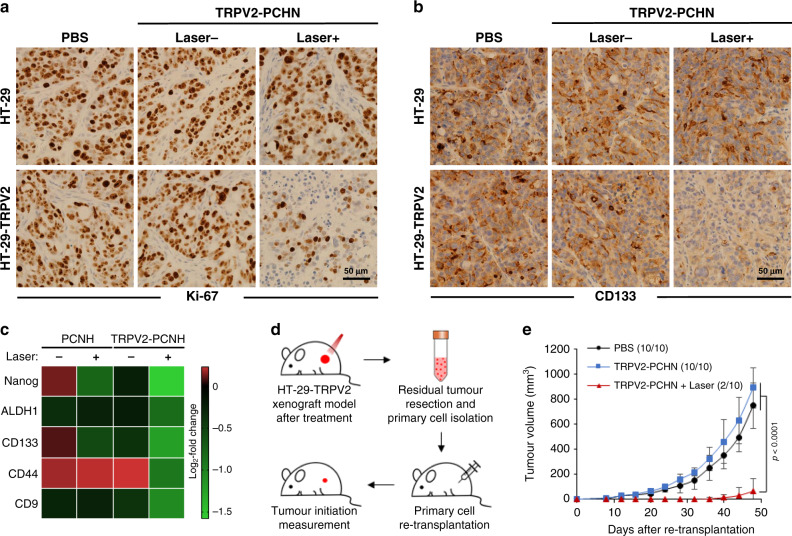
Laser-induced TRPV2-PCNH inhibits tumour re-initiation in in vivo xenograft models with TRPV2 overexpression. **a**, **b** Immunohistochemical stain (IHC) analysis of primary tumour sections. **a** Ki-67 and **b** CD133 expressions were down-regulated by TRPV2-PCNH-mediated NIR treatment in tumour xenografts overexpressing TRPV2. **c** RT-qPCR analysis showing stronger decreases in mRNA levels of stemness-associated markers in TRPV2-overexpressing tumours from the mice treated with TRPV2–PCNH and laser irradiation. **d** De novo tumorigenesis experiment flow (detailed in Methods). **e** Observations of tumour initiation and growth revealed decreases in tumorigenicity of cells isolated from tumours in mice treated with TRPV2–PCNH and laser irradiation (mean ± s.e.m., *n* = 5 biologically independent mice, two-way ANOVA test). 500 cells were injected for each tumour. Tumorigenesis rates are shown in brackets following the legends for each group.

Immunohistochemistry staining of Ki-67 (proliferation marker) and CD133 (stem cell marker) was performed to obtain molecular evidences for the regulation of cancer stemness by laser-driven TRPV2-PCNH nanoparticles. HT-29-TRPV2 tumour tissues exhibited obviously lower expression of both after laser irradiation (Fig. 6a, b). Western blotting revealed downregulation of CD133 in nanocomplexes and laser-treated A549 tumours that were transfected with TRPV2 plasmid in situ (Supplementary Fig. 15). RT-qPCR analyses of tumour tissues from mice treated with PCNH or TRPV2–PCNH nanoparticles revealed decreases in expression levels of stemness-associated markers in the latter following laser irradiation (Fig. 6c). Considering the in vitro inhibitory effects of laser-induced TRRV2–PCNH on cancer cell stemness (Fig. 4), we suggest that TRPV2-targeted phototherapy inhibits re-initiating activities of tumours. To test this hypothesis, we resected HT-29–TRPV2 tumours after treatments, digested them into single-cell cultures and modelled early tumorigenesis by transplanting titrated number of cells into nude mice (Fig. 6d). As shown in Fig. 6e, inoculation of 500 cells from tumours without irradiation (PBS and TRPV2–PCNH groups) resulted in aggressive tumour development with a tumour formation rate of 100%. But primary tumour cells from photoirradiated xenografts were barely transformed (20% tumour formation rate) over 50 days. Comparing to untreated counterpart (PBS group), nanoparticles/laser treatment led to a substantial decrease in fraction of cells with tumour-initiating features (Supplementary Fig. 16). This significant suppression of tumorigenicity of tumour cells could offer a strategy for clinical resolution of cancer metastasis and recurrence. In vivo experiments with A549-TRPV2 xenografts further revealed showed that mice received combination treatment (TRPV2-PCNH mediated laser irradiation followed by PTX drug administration) exhibited strongest suppression in tumour growth as compared to single-approach treatments (Supplementary Fig. 17). Such synergistic effect indicates laser-induced TRPV2-PCNH may improve cancer drug resistance by inhibiting cancer stemness. These observations of phototherapeutic efficacy of TRRV2–PCNH are highly consistent with our in vitro observations.

### Photothermogenetic suppression of Wnt/β-catenin signalling

**Fig. 7:**
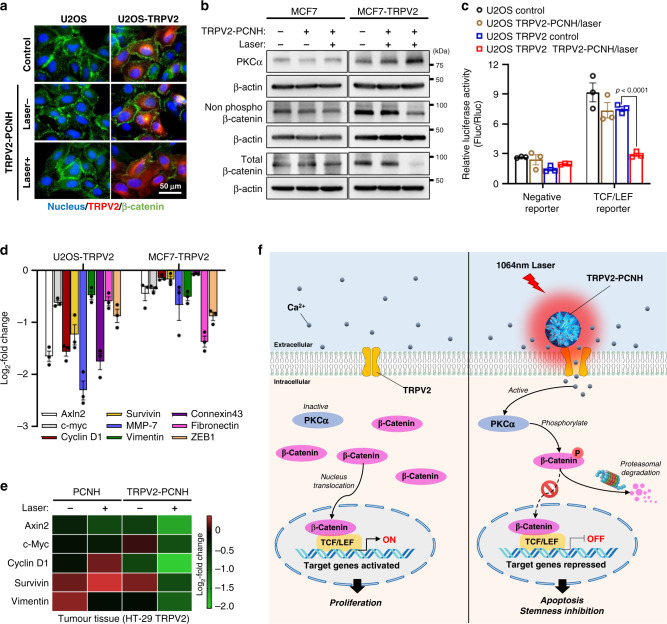
Laser-induced TRPV2–PCNH exerts therapeutic effects by suppressing Wnt/β-catenin signalling. **a** Immunostaining of β-catenin showing decreased expression levels in TRPV2-enriched cells after treatment with nanocomplexes and laser irradiation; blue, Hoechst indicates nuclei; red, mCherry indicates TRPV2; green, Alexa488 indicates β-catenin; **b** Western blotting analyses showed increase in PKCα expression and decreased expression of non-phosphorylated and total β-catenin in nanocomplexes-treated U2OS–TRPV2 cells after irradiation. β-actin was used as an internal control. **c** Luciferase reporter assay driven by β-catenin consensus binding sites (TCF/LEF) showed a decrease in TRPV2-overexpressing U2OS cells after laser-induced TRPV2-PCNH treatment (mean ± s.e.m., *n* = 3 independent experiments, Student’s *t* two-sided test). **d** RT-qPCR analysis of cells overexpressing TRPV2 show that transcript levels of β-catenin target genes were down-regulated after treatments with TRPV2–PCNH nanocomplexes and laser irradiation. Data are represented as means ± standard errors of the mean (s.e.m.); *n* = 3 independent experiments. **e** Transcriptional inhibitions of β-catenin target genes were also observed in tumour tissues after the same treatment. **f** Schematic of the proposed mechanism; in the absence of stimuli (left part), TRPV2 channels are maintained in the off-state to maintain intracellular Ca^2+^ homoeostasis. PKCα is inactivated due to low concentrations of cytosolic Ca^2+^. β-catenin that is stabilised and accumulated in the cytosol translocates into the nucleus and activates its target genes, leading to cancer proliferation. In the presence of stimuli (right part), antibody-guided CNH targets TRPV2 receptors and activates TPRV2 channels through the heat generated from laser radiation. Ca^2+^ influx via TRPV2 channels increases PKCα activity, leading to β-catenin phosphorylation. This phosphorylation promotes rapid degradation of cytosolic β-catenin by the proteasome. Thus, the expression levels of genes that are involved in cell survival and stemness are repressed, resulting in apoptosis and inhibition of cancer stemness.

Wnt/β-catenin signalling has been widely associated with Ca^2+^ mediated cancer cell survival and stemness regulation^35,36^. Herein, we present immunofluorescence experiments showing changes in β-catenin expression following phototherapy. As shown in Fig. 7a, laser irradiation of TRPV2-overexpressing cells treated with nanoparticles resulted in decreased β-catenin expression. Stabilisation of β-catenin is critical for controlling tumorigenesis, and associations of aberrant activated Wnt/β-catenin signalling with carcinogenesis are abundantly documented^37^. Therefore, blockade of β-catenin by TRPV2–PCNH mediated phototherapy offers an attractive mechanism-based therapeutic strategy for cancer treatment.

To better characterise the mechanisms by which TRPV2-guided photothermogentic treatment inhibits β-catenin, we examined photothermal effects on PKCα, which is a protein kinase that directly phosphorylates β-catenin in the presence of Ca^2+^ and promotes its degradation^38^. Western blotting analyses showed increased PKCα expression levels in TRPV2-transfected cells after phototherapy (Fig. 7b). Simultaneously, expression levels of non-phosphorylated (stabilised form) and total β-catenin in these cells were decreased. In contrast, these protein levels were unaffected in cells without TRPV2 transfection after same treatment (Fig. 7b). Moreover, in western blotting and IHC analyses of HT-29-TRPV2 tumour tissues, expression of both non-phospho and total β-catenin were down-regulated only in mice treated with TRPV2-targeted phototherapy (Supplementary Fig. 18). Similar results were also confirmed by IHC experiment using Colon-26 control and TRPV2 overexpressing tumours (Supplementary Fig. 19). Because translocation of β-catenin from the plasma membrane to the nuclear compartment is essential for stem cell regulation and tumorigenesis^39^, we tested if TRPV2-PCNH mediated phototherapy plays a role in β-catenin nuclear localization. Surprisingly, decreased expression level of β-catenin was found in both cytoplasm and nucleus of U2OS-TRPV2 cells after treatment (Supplementary Fig. 20). It is likely attributed to β-catenin upstream regulators that were somehow affected by this photo-treatment, resulting in a substantial decline in the total β-catenin expression (Fig. 7b). In further analyses, β-catenin-dependent luciferase reporter assays showed that laser-induced nanocomplexes caused a significant decrease in TCF/LEF activity in TRPV2 harbouring U2OS cells (Fig. 7c). Consistent with the inactivated β-catenin function, RT-qPCR analyses of downstream targets of β-catenin signalling pathway also showed that, after laser irradiation, all target genes were significantly down-regulated in TRPV2 overexpressing cells and tumours pretreated with TRPV2–PCNH nanoparticles (Fig. 7d, e). The present data demonstrate that Ca^2+^ influx induced by TRPV2–PCNH mediated photothermogenetic therapy activates PKCα, leading to inhibition of Wnt/β-catenin signalling and its target genes.

## Discussion

Tumours are often heterogenous and comprise multiple tumour cell types with varying tumorigenic capacities^40^. CSC theory assumes that small proportions of CSCs in tumours nourish tumour growth^41^. This theory is supported by clinical observations of frequent tumour recurrence in only months or years after tumour resection and chemotherapy, generally presenting as metastases. CSCs have been identified in many common cancer types, including glioma, melanoma, leukaemia, colorectal cancer and breast cancer^42^. In addition to conventional CSC inhibitors, various nanomedicines have been developed to target CSCs^43,44^. Due to the plasticity of CSCs, cancer cells are capable of dynamic phenotypic transitions between non-CSC and CSC states in response to certain stimuli^45^. Thus, the design of innovative treatment strategies that reduce tumour bulk and selectively exterminate CSCs would be most welcome. Herein, we present a photothermogenetic approach using light-driven TRPV2-PCNH, and show promising induction of apoptosis in cancer cells and repression of CSC characteristics. This approach may meet the demands of dual actions.

Ca^2+^ is a ubiquitous cellular signalling molecule that determines cell behaviours by activating or inhibiting cellular signalling cascades and Ca^2+^-regulated proteins^46,47^. Because Ca^2+^ controls pathways of proliferation and apoptosis, treatments that modulate Ca^2+^ homoeostasis may offer efficacious therapies for cancer. Moreover, in contrast with expressed potential drug targets, Ca^2+^ channels, such as those of the TRP family, have altered expression in cancer cells but highly restricted tissue distributions^48^. TRPV2 is overexpressed in bladder cancer cells, and its physiological expression is largely restricted to bone marrow and liver tissues^49^. Hence, treatments that target such molecules with limited tissue distributions are less likely to be associated with generalised side effects. Current techniques for remote stimulation of Ca^2+^ channels are still conceptual, and therapeutic applications remain limited^17,20,21,50–53^. In addition, few studies have addressed specificity, and follow up evidence for tumour inhibition after remission is lacking. We show that TRPV2 antibody-functionalised PCNH nanoparticles caused transient changes in intracellular Ca^2+^ concentrations by photothermal activating TRPV2 ion channels. We also confirm the specificity of the nanoparticles to TRPV2 positive cells with multiple lines of evidence in vitro and in vivo. In addition to evoking apoptosis in cancer cells, our engineered nanocomplexes suppressed cancer cell stemness characteristics after exposure to 1064-nm NIR-II laser irradiation, thus inhibiting tumour re-initiation and abrogating tumour progression. Subsequent molecular analyses revealed that cellular events following excessive Ca^2+^ influx are in part dependent on suppression of Wnt/β-catenin signalling (Fig. 7f) which is frequently activated in human cancers^54^. Besides cancer stemness, there are also evidences to suggest that intrinsic activation of Wnt/β-catenin signalling in cancer cells is associated with T cells deficiency in the tumour microenvironment^55^. Numerous studies demonstrated that inhibition of β-catenin activity could help to reestablish anticancer immunity^56–59^. Indeed, we found the expression level of β-catenin was significantly down-regulated in immunocompetent xenograft models by TRPV2-PCNH-guided phototherapy (Supplementary Fig. 19), suggesting this treatment may transition the tumour microenvironment to a less resistant milieu. Continued studies would accentuate the potential of this technique in combination with immuno- or chemotherapies for achieving a better cancer treatment efficacy.

Collectively, the data presented herein demonstrates the potential of nanocarbon complexes as an optical anticancer agent that inhibit the development of cancer cell stemness. This study contributes to the development of next-generation nanomaterials based optical technologies for the effective treatment of cancers via photothermogenetic activation of ion channels.

## Methods

### Synthesis of TRPV2–PCNH

CNH (average diameter, ca. 80–100 nm; purity, 95%; metal-free) was kindly supplied by NEC Corporation (Tokyo, Japan). TRPV2–PCNH complexes were prepared as follows: 10 mg of CNH and 10 mg of 3-(N-succinimidyloxyglutaryl)aminopropyl, polyethyleneglycol-carbamyl distearoylphosphatidyl-ethanolamine (DSPE–PEG–NHS; SUNBRIGHT DSPE–020GS; Yuka Sangyo, Tokyo, Japan) were dissolved in 9 ml of PBS and were subjected to pulse-type sonication (VCX-600; Sonics, Danbury, CT, USA) for 10 min. The resulting PEGylted CNH (PCNH) solutions were then mixed with 1-ml aliquots of TRPV2 antibody (200 µg in PBS) at 4 °C overnight and then purified by washing. The resulting bionanoconjugates were re-dispersed in 10-ml aliquots of PBS and centrifuged at 653 x *g* for 10 min at 4 °C (model no. 3740; Kubota, Tokyo, Japan). TRPV2–PCNH in supernatants with CNH concentrations of about 1 mg ml^−1^ was collected and used for experiments. Fluorescent TRPV2-PCNH nanocomplexes were generated by conjugating a FITC-labelled TPRV2 antibody. The same protocol was used to prepare IgG–PCNH, except that normal IgG was used instead of TRPV2 antibody. ICG-incorporated TPRV2–PCNH was prepared accordingly using 5 mg of ICG (MP Biomedicals, Tokyo, Japan), 5 mg of CNH and 10 mg of DSPE–PEG–NHS for PEGylation. Cy5-PCNH was obtained in the same manner except that ICG and DSPE-PEG-NHS were replaced with DSPE-PEG2000-Cy5 (Nanosoft Polymers, Winston-Salem, NC, USA).

### Optical and structural characterisations of TRPV2–PCNH

Absorption spectra of TRPV2–PCNH solutions were recorded at room temperature using a UV–Vis–NIR spectrophotometer (V-730 BIO; Jasco, Tokyo, Japan). Hydrodynamic diameters of the PCNH and TRPV2–PCNH complexes were determined using DLS (Photal FPAR-1000; Otsuka Electronics, Osaka, Japan). Negative staining was used to observe morphology and structure of TRPV2–PCNH molecules with a high-resolution TEM (EM-002B; Topcon, Tokyo, Japan) at an acceleration voltage of 80 kV.

### SDS–PAGE analysis of TRPV2–PCNH

IgG–PCNH nanocomplexes and their individual constituents were subjected to standard SDS–PAGE using a Laemmli buffer system. Briefly, 30 µl of IgG–PCNH (1 mg ml^−1^) containing ~10 µg of normal IgG antibody and equivalent constituents were mixed with Laemmli sample buffer (Bio-Rad, Tokyo, Japan), were denatured by heating at 95 °C for 5 min and then loaded onto 12% polyacrylamide gels. Following electrophoresis, gels were stained with colloidal Coomassie brilliant blue (CBB G250; Bio-Rad) for 1 h at room temperature and were then washed with de-staining solution several times. Images were captured using a Gel Doc XR + imaging system (Bio-Rad).

### Photothermal conversion tests

PCNH dispersions and PBS solutions were irradiated with a 1064-nm NIR laser at 1 W (~50 mW mm^−2^; spot diameter, about 5 mm; LSR4064H-3W; Civil Laser, Hangzhou, Zhejiang, China) under the indicated conditions. Temperatures of solutions were measured in real time using a temperature sensor (AD-5601A; A&D, Tokyo, Japan). Thermographic images were recorded using infrared (IR) thermography (i7; FLIR, Nashua, NH, USA).

### Construction of TRPV2-mCherry plasmid

To construct N-terminal mCherry fusion TRPV2 plasmid, the mCherry sequence was amplified using a 5ʼ primer with an EcoRI site (ACTAGAATTCATGGTGAGCAAGGGCGAGGAGGAT) and a 3ʼ primer with a BglII site (GAATAGATCTCTTGTACAGCTCGTCCATGCCGCC). Product fragments were digested with corresponding enzymes and were introduced into a pCMV6-Entry vector containing the full length human TRPV2 coding region (RC202821, OriGene Technologies, Inc., Rockville, MD, USA).

### Cell culture, transfection and treatment

Human bone osteosarcoma (U2OS), lung carcinoma (A549) and normal diploid fibroblasts (TIG3) were obtained from the Japanese Collection of Research Bioresources Cell Bank (Tokyo, Japan). Breast adenocarcinoma (MCF7) and colorectal adenocarcinoma (HT-29) cells were purchased from DS Pharma Biomedical (Tokyo, Japan). Murine colon carcinoma (Colon-26) and rat brain glioma (C6) cells were obtained from the Cell Resource Centre for Biomedical Research, Tohoku University (Sendai, Miyagi, Japan). All cell lines were cultured in Dulbecco’s Modified Eagle’s Medium or Roswell Park Memorial Institute 1640 Medium (Gibco, Grand Island, NY, USA) containing 10% foetal bovine serum, 2-mM l-glutamine, 1-mM sodium pyruvate, gentamycin, penicillin-streptomycin (100 IU ml^−1^) and Hank’s balanced salt solution (Life Technologies, Carlsbad, CA, USA). Cells were maintained at 37 °C in a humidified chamber containing 5% CO_2_ and were cryopreserved in multiple vials in liquid nitrogen. Cell stocks were regularly revived to avoid the genetic instabilities associated with high passage numbers. Transfections were performed with either Lipofectamine 3000 (Life Technologies, NY, USA) or Fugene HD (Roche Applied Sciences, Basel, Switzerland) according to the manufacturers’ instructions. Transfected cells were selected and maintained in medium supplemented with G418. Unless mentioned elsewhere, cells were incubated with TRPV2–PCNH nanocomplexes (50 µg ml^−1^) for 24 h and were then laser irradiated for 90 s. Subsequent analyses were performed 24 h later.

### Cell viability assays

Cell viability was assessed using Cell Counting Kit (CCK)-8 (Dojindo Laboratories, Kumamoto, Japan) according to the manufacturer’s instructions. Briefly, cells (5 × 10^3^ cells well^−1^) were seeded in 96-well plates and were allowed to adhere overnight. Cells were then exposed to nanocomplexes and laser irradiation as indicated. After washing with fresh medium, cells were incubated with CCK-8 solution for 2 h at 37 °C. Absorbance at 450/690 nm was then determined using a microplate reader (Infinite M200 PRO; Tecan, Männedorf, Switzerland).

### Colony formation assays

Cells were treated and irradiated in 96-well plates and were then re-seeded into 6-well plates at a density of 500 cells well^−1^. After attachment, cells were cultured in fresh medium for around 2 weeks. Forming colonies were then washed in cold PBS and were fixed with a pre-chilled methanol/acetone (v/v, 1:1) mixture for 10 min. Fixed cells were stained overnight with 0.1% crystal violet solution (Wako, Osaka, Japan). Numbers of colonies were counted and mean numbers of colonies were calculated from three independent experiments.

### Mammosphere formation assays

MCF-7 and MCF7–TRPV2 cells were initially exposed to laser-induced TRPV2–CNH and were then allowed to recover overnight. Afterwards, control and treated cells were plated at 1 × 10^5^ cells well^−1^ in 6-well ultralow attachment plates (Corning, NY, USA) and were incubated without disturbing in serum-free culture medium (MammoCult™ Human Medium Kit; STEMCELL Technologies, Cambridge, MA, USA) at 37 °C in a humidified atmosphere containing 5% CO_2_. Numbers of spheres with diameters of 50–100 μm and ≥100 μm were evaluated in each well under a microscope on day 7. Mammosphere forming efficiency was calculated as the number of spheres divided by the original number of seeded cells. Experiments were performed three times independently and data are expressed as percentage means ± standard deviations (SD).

### Extreme limiting dilution sphere formation assay

Single-cell suspensions of MCF7 or MCF7-TRPV2 cells were obtained by passing cells through a 40 μm filter. Cells were then seeded at low densities (1, 5, 10, 50, 100 and 500 cells well^−1^) in a 96-well ultralow attached plate with stem cell medium (MammoCult™ Human Medium Kit) at a final volume of 200 μl per well. Each condition was replicated at least in 24 wells. After 14 days, the formation of sphere and their diameter was assessed. Only spheroid bigger than 50 μm in size were included in the analysis. The frequency of initiation capacity was then calculated using Extreme Limiting Dilution Analysis (ELDA).

### Fluorescence microscopy imaging

Cells (1 × 10^5^ cells well^−1^) were plated on glass coverslips in 12-well culture dishes. FITC-labelled TRPV2–PCNH (100 µg ml^−1^) was added when cells had attached to the substratum. After 24-h incubation, cells were washed with PBS and fixed with a methanol/acetone (v/v, 1:1) mixture for 10 min, and nuclei were then stained with Hoechst 33342 (1 µg ml^−1^; Thermo Fisher Scientific, Waltham, MA, USA) for 10 min. After serially washing with PBS, coverslips were mounted for microscope imaging (Axiovert 200 M; Carl Zeiss, Tokyo, Japan).

For immunofluorescence staining experiments, cells were fixed with 4% formaldehyde in PBS for 10 min, were permeabilised in 0.5% Triton X-100 in PBS for 10 min, were blocked in 2% bovine serum albumin in PBS for 1 h and were then incubated with specific primary antibodies (detailed in Supplementary Table 1) overnight at 4 °C. After washing with 0.2% Triton X-100 in PBS (PBS-T), cells were incubated with Alexa Fluor conjugated secondary antibodies for 1 h at room temperature. Counterstaining was performed using Hoechst 33342 following extensive washing with PBS-T. Coverslips with stained cells were finally mounted and stored at 4 °C in the dark until imaging.

Caspase-3 activities were detected using NucView® 488 Caspase-3 Assay Kits (Biotium, Hayward, CA, USA) according to the manufacturer’s instructions. Briefly, live cells were incubated in medium containing caspase-3 substrate (5 µM) for 2 h, and nuclear staining was then performed with Hoechst 33342 for 10 min. After washing with PBS, cells that remained in RPMI 1640 Phenol Red-free medium (Thermo Fisher Scientific) were subjected to live cell imaging using a fluorescence microscope (IX73; Olympus, Tokyo, Japan).

### Measurements of intracellular calcium

Intracellular calcium imaging was performed using non-wash calcium assay Fluo8 kits (AAT Bioquest, Sunnyvale, CA, USA). After 24-h treatments with TRPV2-PCNH (100 µg ml^−1^), cells were washed with PBS, stained with calcium dye and irradiated with a 1064-nm NIR laser (LM164-5W; OptoSigma, Tokyo, Japan) that was incorporated into the fluorescence microscopy system (IX73). Green fluorescence of Fluo-8 from laser-targeted cells was monitored in real-time using an EM-CCD camera (DP80; Olympus). Fluorescence intensities were analysed using ImageJ software (National Institute of Health, Bethesda, MD, USA).

### Flow cytometric analyses

Apoptotic cells were detected using Annexin-V and 7-aminoactinomycin (7-AAD) double staining^60^. After treatments, attached and suspended cells were harvested and numbers of apoptotic cells were determined using a Guava PCA flow cytometer (Millipore) with Guava Nexin Reagent (Millipore) as described by the manufacturer.

AldeRed ALDH detection assays (Millipore) were used to identify populations of cells with high aldehyde dehydrogenase (ALDH) activity. Briefly, control and treated cells were collected and stained with AldeRed-588 substrate at 37 °C for 30 min. Non-treated cells were instead incubated with 50-mM diethylaminobenzaldehyde (DEAB) as a negative control. Labelled cells were then washed and analysed using a Cytomics FC500 flow cytometer (Beckman Coulter, Tokyo, Japan),

CD44 and CD24 expression was detected after washing detached cells with PBS and resuspending in staining buffer (eBioscience™, Thermo Fisher Scientific). Combinations of fluorochrome-conjugated antibodies against human CD44 (PerCP-Cy5.5) and CD24 (PE) or their respective isotype controls (detailed in Supplementary Table 1) were added to cell suspensions at recommended concentrations and were incubated at 4 °C in the dark for 30 min. Analyses were performed on a Cytomics FC500 flow cytometer (Beckman Coulter).

Fractions of apoptotic, ALDH-positive and CD44+/24− cells and mean fluorescence intensity were determined using FlowJo v10 software (Tree Star, Ashland, OR, USA).

### RT-qPCR

Total RNA was isolated from cells and tumour tissues using RNeasy mini kits (Qiagen, Standford Valencia, CA, USA). Concentrations and purities of RNA samples were determined by ultraviolet spectrophotometry using a NanoDrop ND-1000 (Nanodrop Technologies, Wilmington, DE, USA) instrument. Equal amounts of RNA were used for reverse transcription according to the protocol of the QuantiTect Reverse Transcription Kit (Qiagen). Real-time RT-qPCR was then performed using SYBR Select Master Mix (Applied Biosystems, Thermo Fisher Scientific) in triplicate on an Eco™ real-time system (Illumina, San Diego, CA USA). Relative mRNA expression was normalised against that of 18S using the ΔC_t_ method. Primer sets are listed in Supplementary Table 2. Experiments were performed at least thrice.

### Dual luciferase reporter assay

TCF/LEF reporter kit (Cignal report; Qiagen) was employed to evaluate Wnt/β-catenin signalling activity according to the manufacturer’s instructions. Briefly, cells were transiently transfected in triplicate with TCF/LEF luciferase reporters by use of Fugene HD (Roche). Luciferase activity was measured at 48 h after transfection with the Dual-luciferase reporter assay system (Promega, WI, USA). The firefly luciferase activity was normalized against the Renilla luciferase activity. Experiments were performed at least three times.

### Western blotting analyses

Cells and tissues were lysed in radio-immune precipitation assay buffer (Thermo Fisher Scientific) containing complete protease inhibitor cocktail (Roche Applied Science). Nuclear and cytoplasmic fractions were prepared using the NE-PER® Nuclear and Cytoplasmic Extraction reagents (Thermo Fisher Scientific) as per manufacturer’s instruction. Protein concentrations were determined using Pierce Bicinchoninic Acid Protein Assay kits (Thermo Fisher Scientific). Protein lysates (20 μg) were resolved in SDS-polyacrylamide gels, were transferred to polyvinylidene difluoride (PVDF) membranes and were then probed with primary antibodies. PVDF membranes were then incubated with respective secondary antibodies and immunolabeled proteins were imaged using a Gel Doc XR + system (Bio-Rad). Pixels in western blot bands were counted and normalised to those in actin bands (internal loading control) using ImageJ software (National Institute of Health). Antibody details and their dilutions are listed in Supplementary Table 1.

### Animal and tumour models

All animal experimental procedures were approved by the Institutional Animal Care and Use Committee of JAIST and AIST. Female BALB/cSlc (4 weeks old; average weight, 16 g) and BALB/cSlc-nu/nu mice (4 weeks old; average weight, 18 g) were purchased from Japan SLC (Hamamatsu, Japan) and were housed in specific pathogen-free facilities with a 12-h light/12-h dark cycle and free access to food and water.

To generate tumour models, nude mice were subcutaneously inoculated in the flanks with equivalent number of cells (5 × 10^5^ cells for HT-29; 1 × 10^6^ cells for A549 and Colon-26) suspended in 100-μl aliquots of culture medium/Matrigel (Corning) mixture (v:v = 1:1). For in vivo tumour phototherapy experiments, mice were bilaterally implanted with either control or TRPV2 overexpressing derivative cells into flanks. For in vivo biodistribution analyses, both cell types were injected into opposite flanks of the same mouse. For in vivo drug and phototherapy combination experiments, only TRPV2-transfected A549 cells were injected into the right flank of mice. Tumour sizes were monitored using vernier calipers and tumour volumes were calculated as V = L × W^2^/2, where L and W denote lengths and widths of tumours, respectively. Tumour models were randomly divided and used in experiments when tumour volumes reached about 100 mm^3^.

### In vivo biodistribution analyses

To assess biodistributions of nanocomplexes, the NIR fluorescence dye ICG was incorporated into TPRV2-PCNH as described above. HT-29 and HT-29-TRPV2 tumour-bearing mice (*n* = 5) were then injected with 200-μl aliquots of ICG-TPRV2-PCNH or ICG-PCNH (equivalent to a total of 5-mg kg^−1^ CNH) via the tail vein. Optical imaging analyses of whole mice and major organs were performed at the indicated time points using an IVIS Imaging Spectrum System (PerkinElmer, MA, USA) with emission at 800 nm and excitation at 740 nm. Fluorescence intensities were quantified using IVIS Living Imaging 3.0 software (PerkinElmer).

### Quantitative pharmacokinetic analyses

Female Jcl:ICR mice (6 weeks old; average weight, 26 g) (Japan SLC) (*n* = 5) were intravenously injected with Cy5-labelled CNH (5 mg kg^−1^) and were killed at 1 h, 24 h, 7 days and 14 days post injection. Blood and organs were weighed and solubilized by RIPA buffer (Thermo Fisher Scientific) using a TissueLyser (Qiagen). The clear homogeneous tissue lysates were diluted 50 times and subjected to Tecan microplate reader for fluorescence intensity measurement. The blood and organs from control mice without injection of nanocomplexes were collected and used as controls to subtract the auto-fluorescence background. A series of dilutions of Cy5-PCNH standard samples were performed to obtain a standard calibration curve for quantitative determination of PCNH. The concentration of the PCNH in the various organs of the mice was calculated and presented as percentage of injected dose per gram of tissue (%ID).

### In vivo transfection

Female BALB/c nude mice bearing A549 tumours randomly divided into groups of five mice each when tumours reached the size of ∼100 mm^3^. 5 μg of TRPV2 DNA plasmid was delivered in vivo by an intratumoral injection with in vivo-jetPEI reagent (Polyplus Transfection, NY, USA), according to the manufacturer’s instructions. Injections were repeated twice a week for next 3–4 weeks during the treatment of PCNH nanocomplexes and laser irradiation.

### In vivo tumour phototherapy

Mice bearing control or TRPV2-overexpressing cell-derived tumours (*n* = 6) were intraperitoneally injected with 200-μl aliquots of PBS, 200-μl aliquots of PBS dispersions containing PCNH (5 mg kg^−1^) or 200-μl aliquots of PBS dispersions containing TRPV2-PCNH (5 mg kg^−1^). Injections were performed every other day. On days as indicated, tumours on the right-side of backs were irradiated with a 1064-nm laser at 1 W (~50 mW mm^−2^) for 5 min. When tumours were larger than the laser spot size (diameter ~5 mm), two or three locations were irradiated. Surface temperatures of irradiated tumours were monitored using IR thermography (i7; FLIR, Nashua, NH, USA). Before mice were sacrificed, tumours were isolated for further analysis as needed. Tumour volumes and overall health (body weight) were recorded every other day for the duration of experiments.

For laser and drug combination therapy, A549-TRPV2 tumour-bearing mice were randomly separated into four groups (5 mice per group). Intraperitoneal injections of nanocomplexes containing paclitaxel (50 mg kg^−1^, dispersed in 10% Cremophor EL (Sigma-Aldrich)), TRPV2-PCNH (50 mg kg^−1^) or their combination were started when small tumour buds were formed (ca. 100 mm^3^). Equivalent volume of PBS was used as negative control. The injections were performed every alternate day. Tumours were irradiated for 5 min using the 1064 nm laser at 1 W (ca. 50 mW mm^−2^) on Day 3 and Day 6 since injection began. The administration of TRPV2-PCNH was also stopped after Day 6. Tumour volumes were recorded until the experiment is completed.

### Immunohistochemistry (IHC) analyses

IHC analysis was performed by New Histo. Science Laboratory (Tokyo, Japan) with standard protocols. Briefly, primary tumours were surgically removed, fixed in 10% formalin, processed for paraffin embedding, and then cut into 3~4-μm-thick sections. After incubation with primary antibodies (listed in Supplementary Table 1) and HRP-labelled polymer, the sections were stained with hematoxylin and then examined by light microscopy (Axiovert 200 M; Carl Zeiss).

### Primary cell isolation and tumour-initiating assays

HT-29–TRPV2 tumours that had been NIR irradiated were resected, washed with PBS and then cut into pieces on ice. Shredded tumours were then next immersed in 2% collagenase A (Roche) and were digested at 37 °C with gentle shaking for 1 h. Dissociated cells were then filtered through 100-µm mesh and were kept in culture medium at 4 °C. To assess tumour-initiating capacity, cells were counted and isolated from each tumour and were subcutaneously re-transplanted into the mice (Female, BALB/cAJc-nu/nu, 6 weeks old; *n* = 5, two tumours per mouse) in diluting concentrations (5 × 10^2^, 5 × 10^3^ and 5 × 10^4^ cells injection^−1^). Tumour volumes were monitored once every 3 days as described above. The frequency of stem cell initiation was analysed using ELDA.

### Statistics and reproducibility

All experiments were performed in triplicate and repeated three or more times. Quantitative values are expressed as the means ± standard error of the mean (SEM), or means ± SD, of at least three independent experiments. Pairwise differences were identified using Student’s *t* test and comparisons of multiple groups were performed using two-way analysis of variance (ANOVA) followed by Tukey test. A *p* value less than 0.05 is considered to be statistically significant.

### Reporting summary

Further information on research design is available in the Nature Research Reporting Summary linked to this article.

## Supplementary information

Supplementary Information

Description of Additional Supplementary Files

Supplementary Movie 1

Supplementary Movie 2

Supplementary Movie 3

Supplementary Movie 4

Supplementary Movie 5

Supplementary Movie 6

Reporting Summary

## Data Availability

All data needed to evaluate the conclusions in the paper are presented in the paper and/or the Supplementary Materials. Additional data related to this paper are available from the corresponding author on reasonable request. A reporting summary for this article is available as a Supplementary Information file.

## References

[1] Laplane, L. (eds) Cancer stem cells: philosophy and therapies (Harvard University Press, Elsevier, 2016).

[2] Adorno-Cruz V (2015). Cancer stem cells: targeting the roots of cancer, seeds of metastasis, and sources of therapy resistance. Cancer Res..

[3] Phi LTH (2018). Cancer stem cells (CSCs) in drug resistance and their therapeutic implications in cancer treatment. Stem Cells Int..

[4] Chen J (2018). Inhibition of cancer stem cell like cells by a synthetic retinoid. Nat. Commun..

[5] Li S (2018). Inhibition of DNMT suppresses the stemness of colorectal cancer cells through down-regulating Wnt signaling pathway. Cell. Signal..

[6] Huang X (2018). The molecular basis for inhibition of stemlike cancer cells by salinomycin. ACS Cent. Sci..

[7] Rivnay J, Wang H, Fenno L, Deisseroth K, Malliaras GG (2017). Next-generation probes, particles, and proteins for neural interfacing. Sci. Adv..

[8] Colombo E, Feyen P, Antognazza MR, Lanzani G, Benfenati F (2016). Nanoparticles: a challenging vehicle for neural stimulation. Front. Neurosci..

[9] Deubner J, Coulon P, Diester I (2019). Optogenetic approaches to study the mammalian brain. Curr. Opin. Struct. Biol..

[10] Ma G (2017). Optogenetic toolkit for precise control of calcium signaling. Cell Calcium.

[11] Stanley SA (2012). Radio-wave heating of iron oxide nanoparticles can regulate plasma glucose in mice. Science.

[12] Stanley SA, Sauer J, Kane RS, Dordick JS, Friedman JM (2015). Remote regulation of glucose homeostasis in mice using genetically encoded nanoparticles. Nat. Med..

[13] Gao W (2018). Copper sulfide nanoparticles as a photothermal switch for TRPV1 signaling to attenuate atherosclerosis. Nat. Commun..

[14] Cho MH (2012). A magnetic switch for the control of cell death signalling in in vitro and in vivo systems. Nat. Mater..

[15] Chechetka SA, Doi M, Pichon BP, Bégin-Colin S, Miyako E (2016). Photothermal and mechanical stimulation of cells via dualfunctional nanohybrids. Nanotechnology.

[16] Miyako E, Chechetka SA, Doi M, Yuba, Kono KE (2015). In vivo remote control of reactions in Caenorhabditis elegans by using supramolecular nanohybrids of carbon nanotubes and liposomes. Angew. Chem. Int. Ed..

[17] Miyako E (2014). Photofunctional nanomodulators for bioexcitation. Angew. Chem. Int. Ed..

[18] Chechetka SA (2016). Magnetically and near-infrared light-powered supramolecular nanotransporters for the remote control of enzymatic reactions. Angew. Chem. Int. Ed..

[19] Miyako E (2012). Photothermic regulation of gene expression triggered by laser-induced carbon nanohorns. Proc. Natl Acad. Sci. USA.

[20] Chechetka SA (2017). Light-driven liquid metal nanotransformers for biomedical theranostics. Nat. Commun..

[21] Lyu Y, Xie C, Chechetka SA, Miyako E, Pu K (2016). Semiconducting polymer nanobioconjugates for targeted photothermal activation of neurons. J. Am. Chem. Soc..

[22] Tahara Y (2011). Histological assessments for toxicity and functionalization-dependent biodistribution of carbon nanohorns. Nanotechnology.

[23] Miyawaki J, Yudasaka M, Azami T, Kubo Y, Iijima S (2008). Toxicity of single-walled carbon nanohorns. ACS Nano.

[24] Zhang M, Yamaguchi T, Iijima S, Yudasaka M (2013). Size-dependent biodistribution of carbon nanohorns in vivo. Nanomedicine.

[25] Kojima I, Nagasawa M (2014). TRPV2. Handb. Exp. Pharmacol..

[26] Marwaha L (2016). TRP channels: potential drug target for neuropathic pain. Inflammopharmacology.

[27] Hu JJ, Cheng YJ, Zhang XZ (2018). Recent advances in nanomaterials for enhanced photothermal therapy of tumors. Nanoscale.

[28] Zhu S, Tian R, Antaris AL, Chen X, Dai H (2019). Near-infrared-II molecular dyes for cancer imaging and surgery. Adv. Mater..

[29] Cong B (2014). Gold nanorods: near-infrared plasmonic photothermal conversion and surface coating. J. Mater. Sci. Chem. Eng..

[30] Sun T (2018). Second near-infrared conjugated polymer nanoparticles for photoacoustic imaging and photothermal therapy. ACS Appl. Mater. Interfaces.

[31] O’Reilly D, Buchanan P (2019). Calcium channels and cancer stem cells. Cell Calcium.

[32] Zhang M (2014). Quantification of whole body and excreted carbon nanohorns intravenously injected into mice. Adv. Healthc. Mater..

[33] Zhang M, Yamaguchi T, Iijima S, Yudasaka M (2013). Size-dependent biodistribution of carbon nanohorns in vivo. Nanomedicine.

[34] Miyawaki J (2009). Biodistribution and ultrastructural localization of single-walled carbon nanohorns determined in vivo with embedded Gd2O3 labels. ACS Nano.

[35] Mattson MP, Chan SL (2003). Calcium orchestrates apoptosis. Nat. Cell Biol..

[36] Fang L (2016). A small-molecule antagonist of the β-catenin/TCF4 interaction blocks the self-renewal of cancer stem cells and suppresses tumorigenesis. Cancer Res..

[37] MacDonald BT, Tamai K, He X (2009). Wnt/beta-catenin signaling: components, mechanisms, and diseases. Dev. Cell.

[38] Gwak J (2006). Protein-kinase-C-mediated beta-catenin phosphorylation negatively regulates the Wnt/beta-catenin pathway. J. Cell Sci..

[39] Clevers H (2006). Wnt/β-catenin signaling in development and disease. Cell.

[40] Clevers H (2016). Cancer therapy: defining stemness. Nature.

[41] Batlle E, Clevers H (2017). Cancer stem cells revisited. Nat. Med..

[42] Klonisch T (2008). Cancer stem cell markers in common cancers - therapeutic implications. Trends Mol. Med..

[43] Liu Y (2015). Gd-metallofullerenol nanomaterial as non-toxic breast cancer stem cell-specific inhibitor. Nat. Commun..

[44] Xu Y (2014). Selective inhibition of breast cancer stem cells by gold nanorods mediated plasmonic hyperthermia. Biomaterials.

[45] Cabrera MC, Hollingsworth RE, Hurt EM (2015). Cancer stem cell plasticity and tumor hierarchy. World J. Stem Cells.

[46] Monteith GR, Prevarskaya N, Roberts-Thomson SJ (2017). The calcium-cancer signalling nexus. Nat. Rev. Cancer.

[47] Clapham DE (2007). Calcium signaling. Cell.

[48] Monteith GR, McAndrew D, Faddy HM, Roberts-Thomson SJ (2007). Calcium and cancer: targeting Ca2+ transport. Nat. Rev. Cancer.

[49] Ouadid-Ahidouch H, Dhennin-Duthille I, Gautier M, Sevestre H, Ahidouch A (2013). TRP channels: diagnostic markers and therapeutic targets for breast cancer?. Trends Mol. Med..

[50] Ma G (2018). Optogenetic control of voltage-gated calcium channels. Angew. Chem. Int. Ed..

[51] Huang H, Delikanli S, Zeng H, Ferkey DM, Pralle A (2010). Remote control of ion channels and neurons through magnetic-field heating of nanoparticles. Nat. Nanotechnol..

[52] Zhen X (2018). Semiconducting photothermal nanoagonist for remote-controlled specific cancer therapy. Nano Lett..

[53] He L (2015). Near-infrared photoactivatable control of Ca(2+) signaling and optogenetic immunomodulation. eLife.

[54] Reya T, Clevers H (2005). Wnt signalling in stem cells and cancer. Nature.

[55] Trujillo JA, Sweis RF, Bao R, Luke JJ (2018). T Cell-inflamed versus non-T cell-inflamed tumors: a conceptual framework for cancer immunotherapy drug development and combination therapy selection. Cancer Immunol. Res..

[56] Yaguchi T (2012). Immune suppression and resistance mediated by constitutive activation of Wnt/β-catenin signaling in human melanoma cells. J. Immunol..

[57] Hong Y (2015). β-Catenin promotes regulatory T-cell responses in tumors by inducing vitamin A metabolism in dendritic cells. Cancer Res..

[58] Wang B, Tian T, Kalland KH, Ke X, Qu Y (2018). Targeting Wnt/β-catenin signaling for cancer immunotherapy. Trends Pharm. Sci..

[59] Galluzzi L, Spranger S, Fuchs E, López-Soto A (2019). WNT signaling in cancer immunosurveillance. Trends Cell Biol..

[60] Yu Y (2017). Withaferin-A kills cancer cells with and without telomerase: chemical, computational and experimental evidences. Cell Death Dis..

