# Supplementary material for: Photothermogenetic inhibition of cancer stemness by near-infrared-light-activatable nanocomplexes
Source: Nat Commun. 2020 Aug 17;11:4117. doi: 10.1038/s41467-020-17768-3 (PMC7431860; doi:10.1038/s41467-020-17768-3)

Supplementary Information for

Photothermogenetic inhibition of cancer stemness by near-infrared-light-activatable nanocomplexes

Yue Yu et al.

Supplementary Figures & Tables

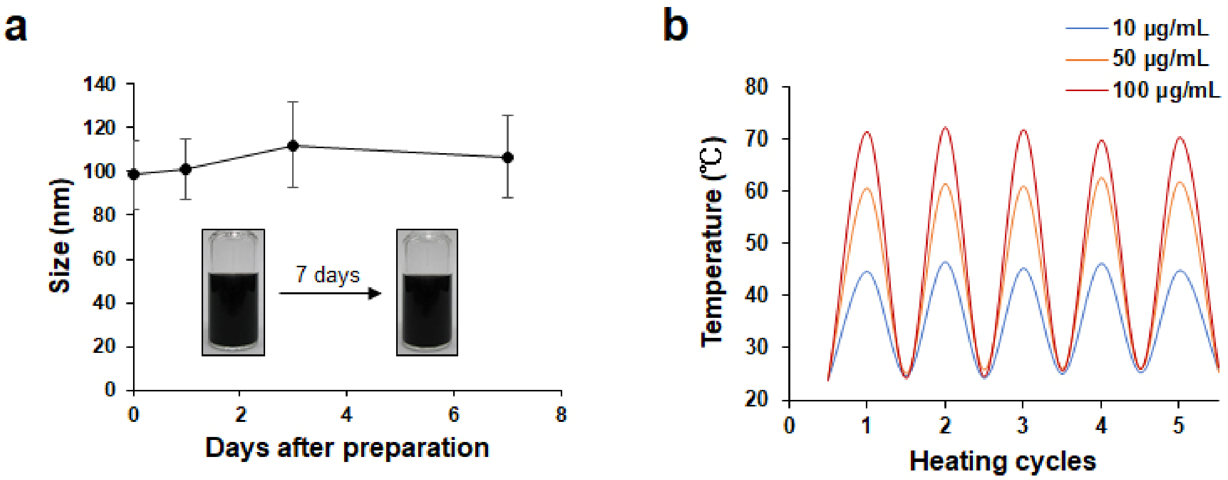

Supplementary Figure 1. (a) Dispersion stability of TRPV2–PCNH in PBS; data represent DLS measurements at 0, 1, 3, and 7 days. Inset images show TRPV2–PCNH solution (100 µg ml^{-1}) before and after standing for 7 days. Data are represented as means ± standard division (s.d.); n = 3 independent experiments. (b) Photothermal stability of TRPV2–PCNH after five on/off cycles of laser irradiation for 3 min; TRPV2–PCNH volume, 200 µL; 1064 nm laser power, 1 W (~50 mW mm^{-2}).

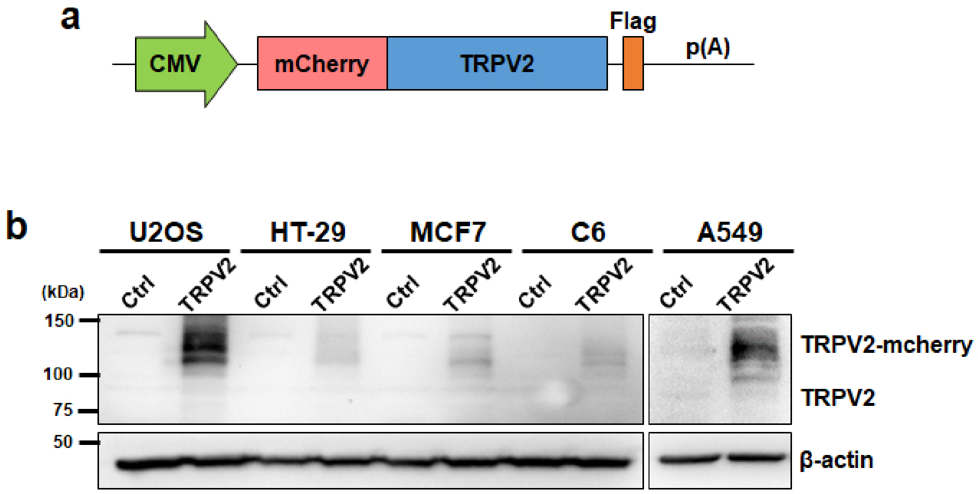

Supplementary Figure 2. (a) Diagrammatic presentation of the TRPV2 expression reporter. The plasmid contained cytomegalovirus (CMV) enhancer to drive the expression of the inserted mCherry fluorescent protein simultaneously with the TRPV2 fusion protein. Myc and DDK flag-tags were added before the polyadenylation sequence [p(A)]. (b) Western blotting of TRPV2 in control and transfected cells shows successful expression of exogenous TRPV2 in these cell lines.

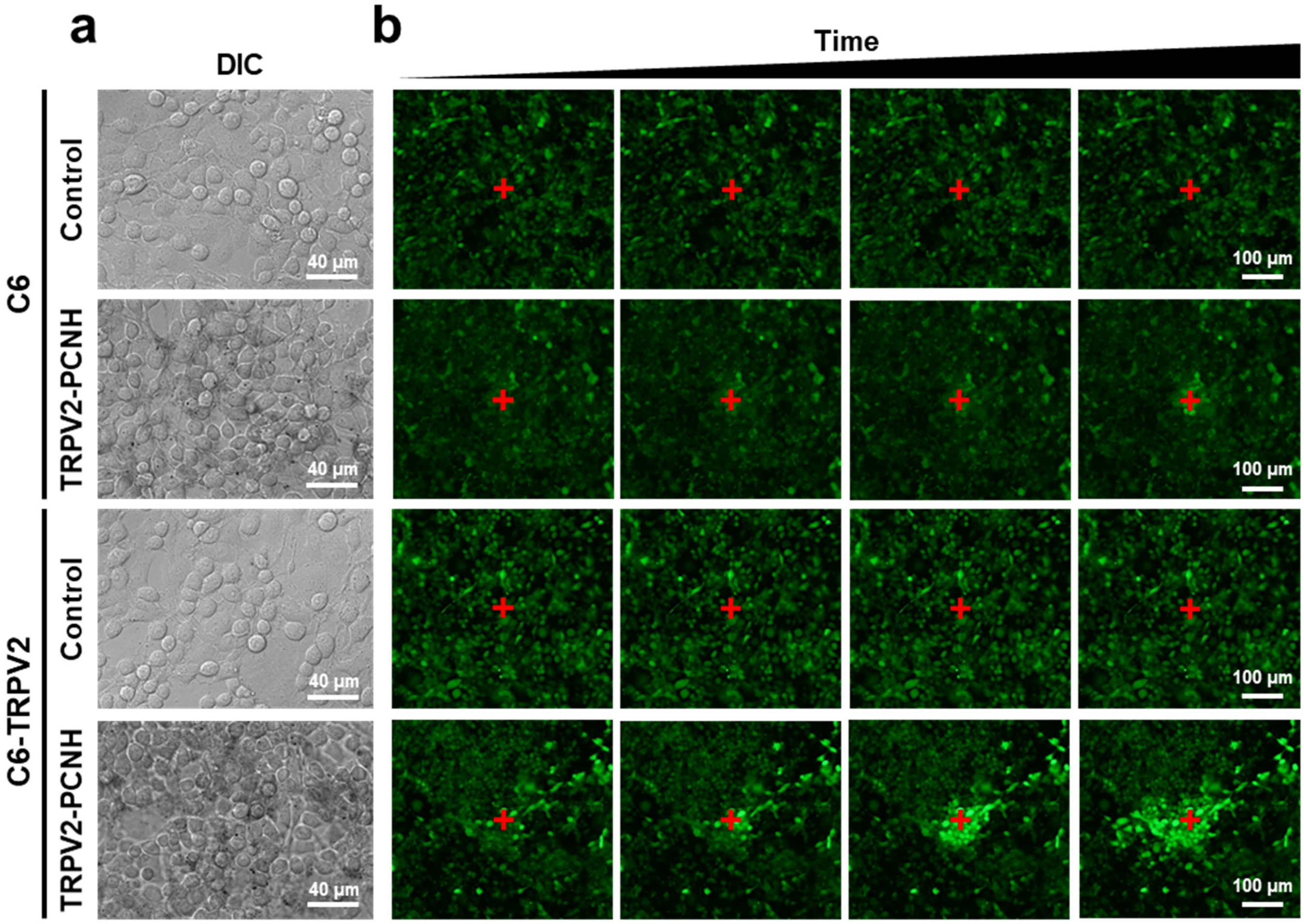

Supplementary Figure 3. (a) DIC microscopy imaging of C6 parental and TRPV2-transfected cells following treatments with TRPV2–PCNH (100 µg ml^{-1}) for 24 h. Nanocomplexes (black dots) were more efficiently internalized by cells overexpressing TRPV2. (b) Time lapse fluorescence images of cells with the same nanocomplex treatments show selective induction of Ca^{2+} influx in TRPV2-transfected cells following laser stimulation at 0.7 W (~97 mW mm^{-2}) for 1 s. Cells were loaded with Ca^{2+} indicator (Green) for 30 min before irradiation. Red crosses indicate irradiated spots.

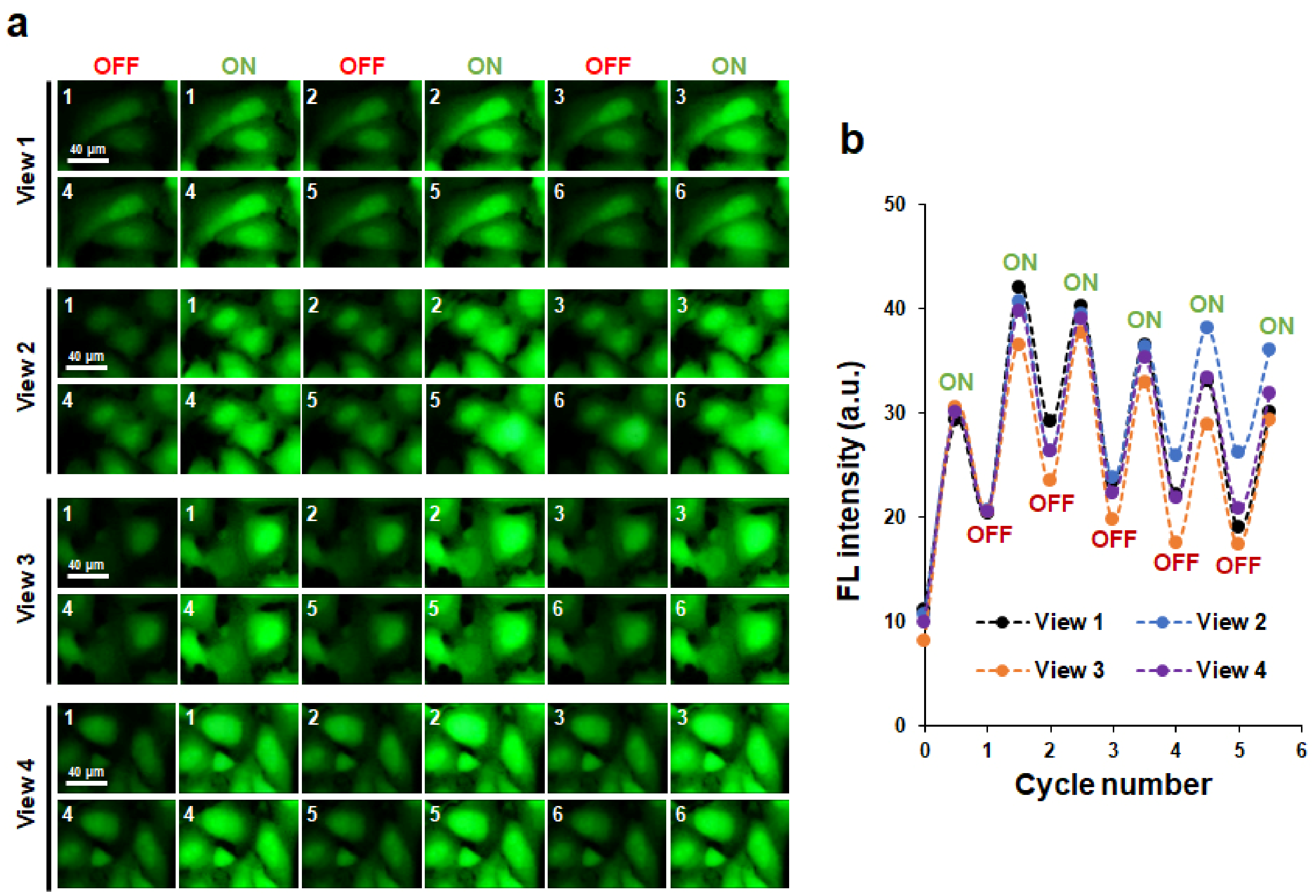

Supplementary Figure 4. (a) Fluorescence images of TRPV2-overexpressing U2OS cells treated with TRPV2–PCNH show reversible induction of Ca^{2+} influx under repeated laser stimulation. Cells were incubated with TRPV2–PCNH (100 μg ml^{-1}) for 24 h and were loaded with Ca^{2+} indicator for 30 min before irradiation (0.7 W, ~97 mW mm^{-2}, 1 s). (b) Quantitation of fluorescence intensities at four different views is shown on the right.

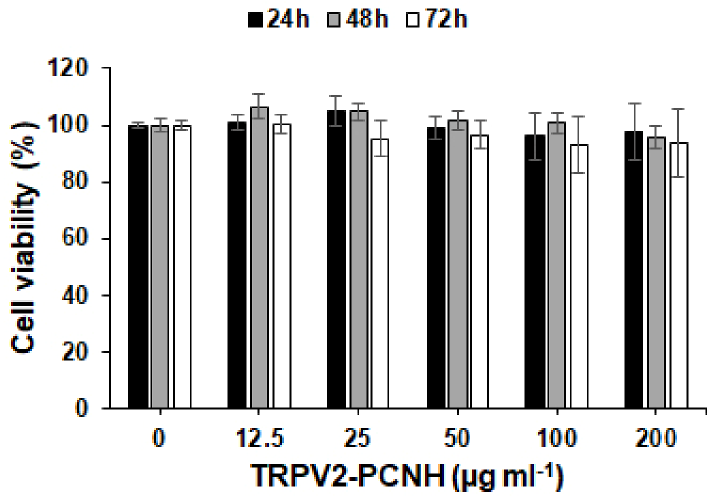

Supplementary Figure 5. Cell viability assays showing TRPV2–PCNH (up to 200 μg ml^{-1}) did not induce toxicity to TIG-3 normal fibroblasts within 72 h of treatment. Data are represented as means ± standard errors of the mean (s.e.m.); n = 3 biologically independent tests.

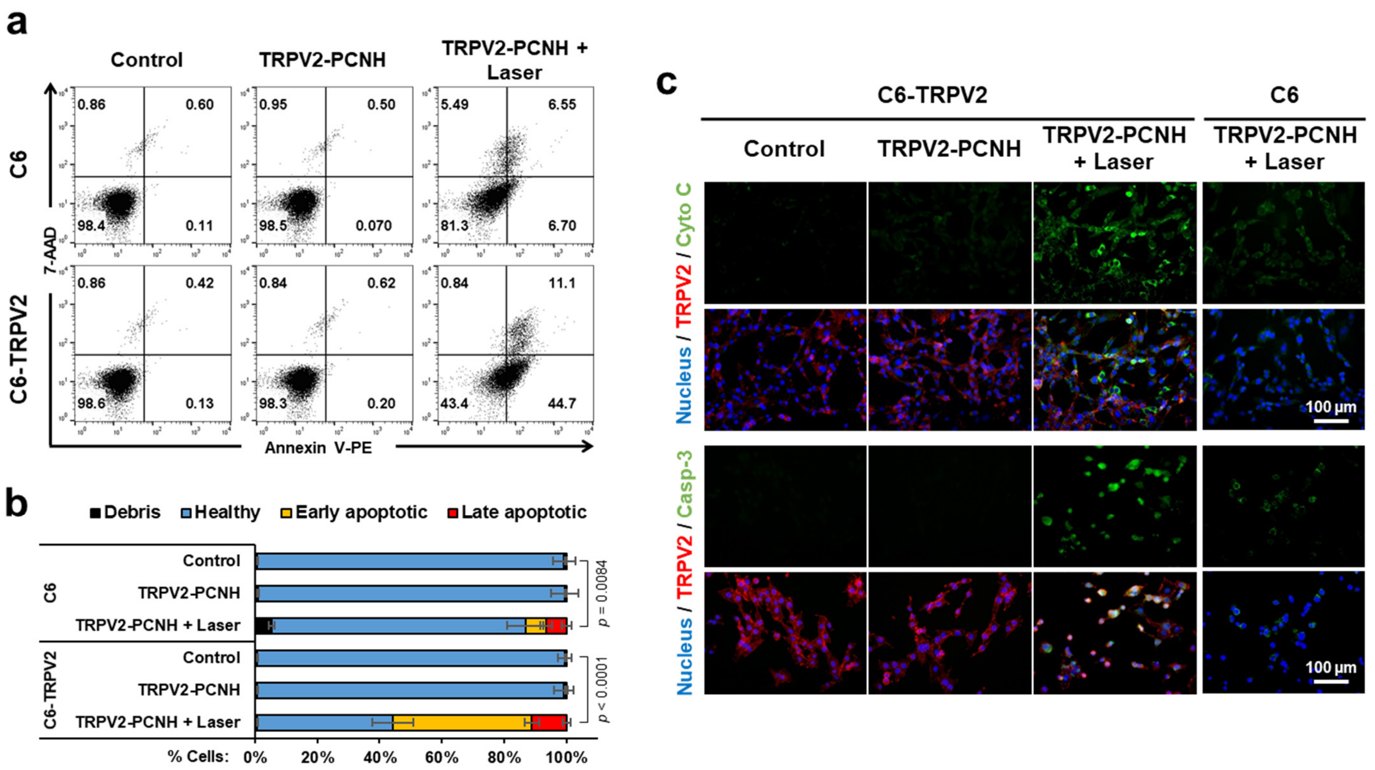

Supplementary Figure 6. (a) Flow cytometric analysis showing stronger apoptosis induction in TRPV2-transfected C6 cells treated with TRPV2–PCNH (50 µg ml^{-1}) and irradiation (1 W, ~50 mW mm^{-2}, 90 s). Measurements were performed at 24 h after irradiation. (b) Quantitation is shown below. Significant differences in total numbers of apoptotic cells (mean ± s.e.m., n = 3 independent experiments) between treated and control cells were identified using Student's two-sided t test. (c) Fluorescence imaging of control and TRPV2-transfected C6 cells showing increased expression of cytochrome c and caspase-3 after treatment with TRPV2–PCNH (50 µg ml^{-1}) and laser irradiation (1 W, ~50 mW mm^{-2}, 90 s); blue, Hoechest indicates Nuclei; red, mCherry indicates TRPV2; green, Alexa488 indicates cytochrome c, and NucView488 indicates caspase-3.

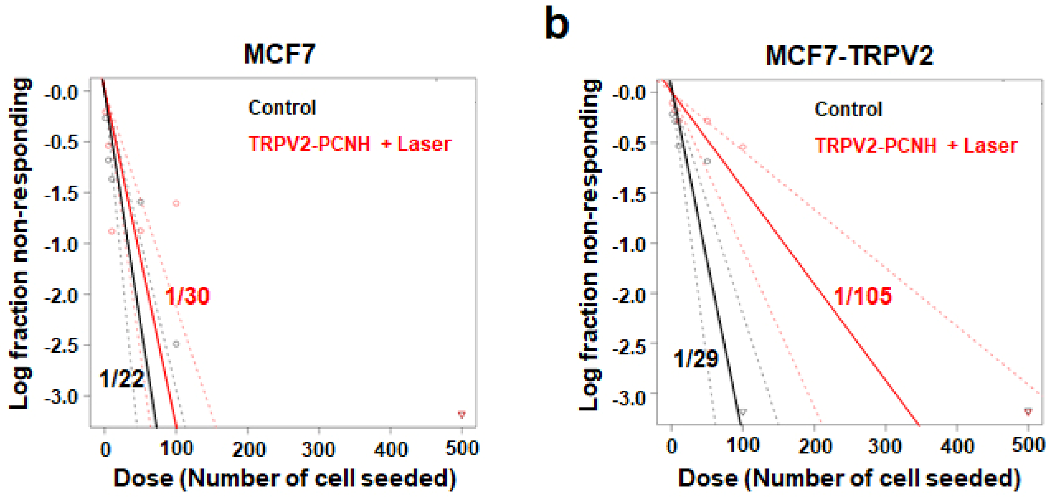

Supplementary Figure 7. Extreme sphere limiting dilution analyses comparing sphere forming frequency between control and TRPV2-PCNH/laser treated (a) MCF7 and (b) MCF7-TRPV2 cells. The cells were seeded at a density of 500 cells/well to 1 cell/well and cultured for 14 days.

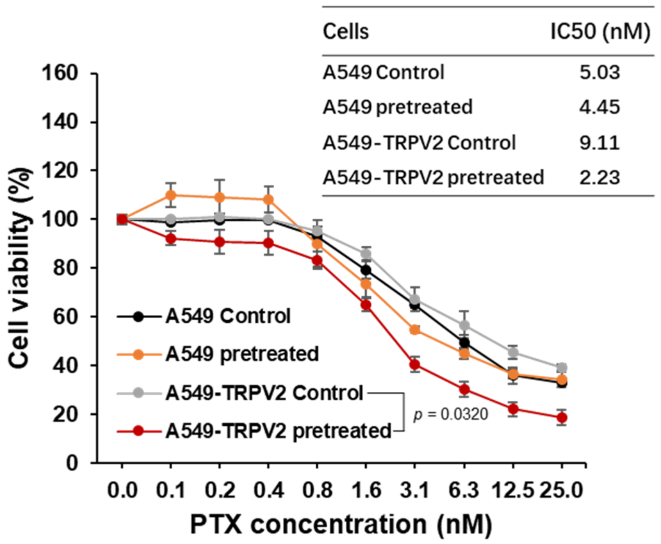

Supplementary Figure 8. Sensitivity of A549 control and TRPV2-overexpressing cells to PTX anticancer drug (mean ± s.e.m., n = 3 biologically independent experiments, two-way ANOVA test). Cells were pretreated with or without TPRV2-PCNH mediated photo-stimulation (50 µg ml^{-1} of TRPV2-PCNH; Laser irradiation at 1 W for 90 sec). Cell viability was measured at 48 h post drug exposure. IC50 values of each group are represented in the upper right corner.

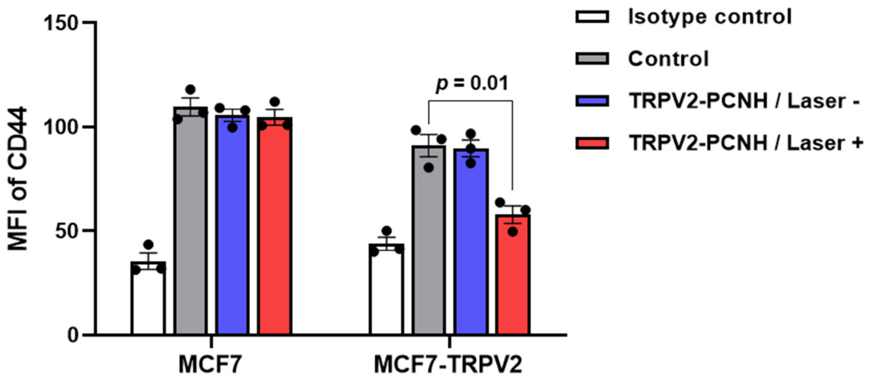

Supplementary Figure 9. Mean fluorescence intensity (MFI) of MCF7 and TRPV2-MCF7 cells treated with or without TRPV2-PCNH nanocomplexes and laser irradiation (mean ± s.e.m., n = 3 independent experiments), P value was defined using Student's two-sided t test.

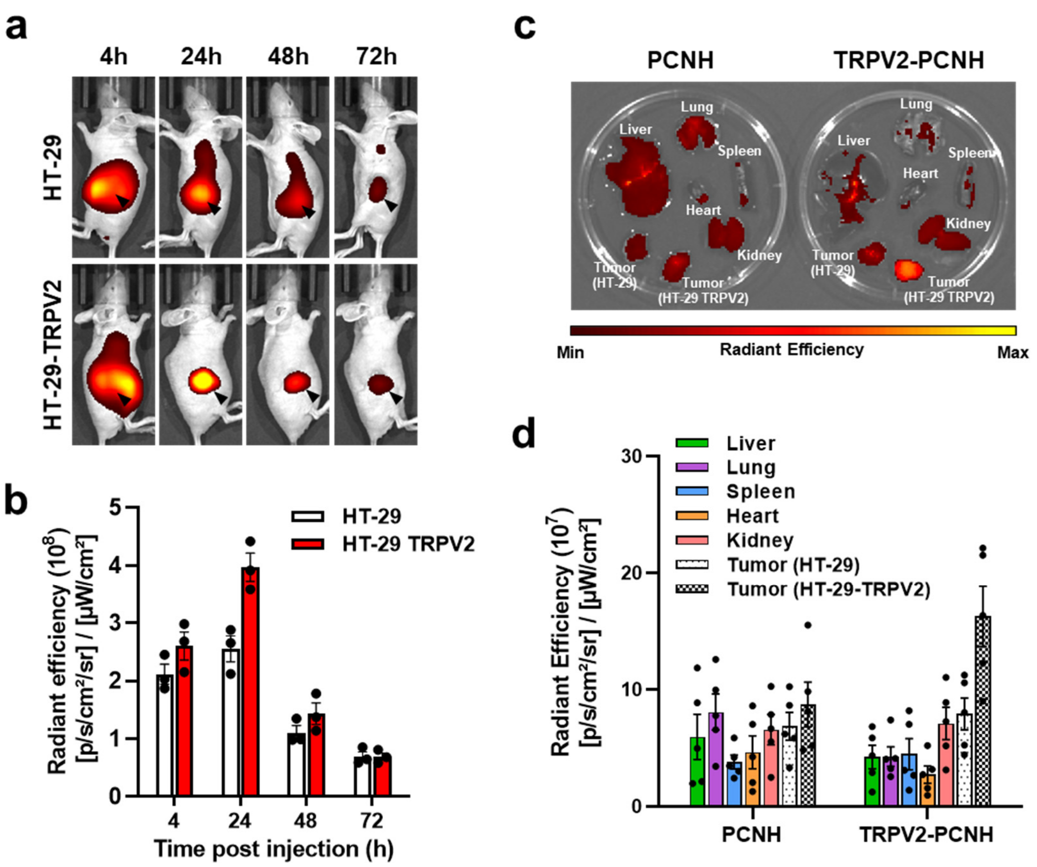

Supplementary Figure 10. (a) Fluorescence imaging of tumor-bearing mice (n = 5) after intravenous injections of ICG-labeled TRPV2–PCNH (5 mg kg^{-1}); HT-29 control and TRPV2 overexpressing cells were implanted subcutaneously into the left and right flanks of the same mouse, respectively. Black arrows indicate tumor sites. (b) Corresponding radiant efficiencies of tumors at different times after injections are shown below. Data are represented as means ± s.e.m.; n = 5 biologically independent tumours; (c) *Ex vivo* imaging and (d) quantitative radiant efficiency of tumors and major organs at 24 h after injections. Data are represented as means ± s.e.m.; n = 5 biologically independent mice.

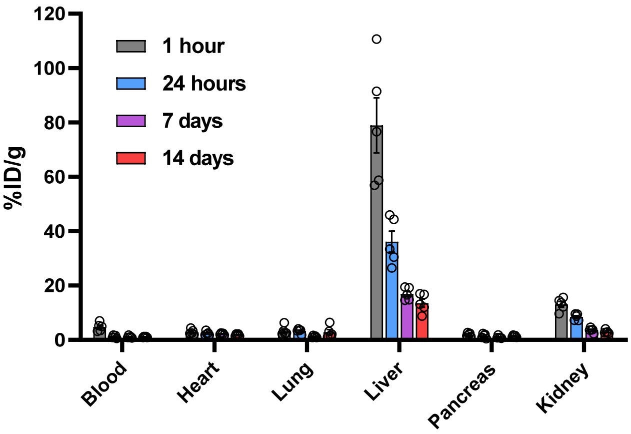

Supplementary Figure 11. Quantitative pharmacokinetic analysis of CNHs in mice by measuring the Cy5 fluorescence intensity in the blood and major organs at different time points post-injection. Data are represented as means ± s.e.m.; n = 5 biologically independent mice.

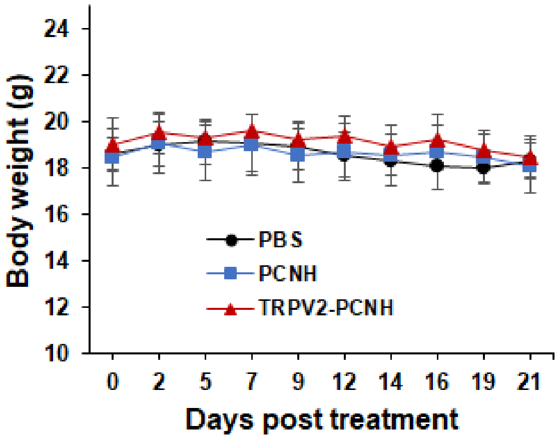

Supplementary Figure 12. Average body weights of mice during treatments with PBS, PCNH, and TRPV2–PCNH. Data are represented as means ± s.e.m.; n = 6 biologically independent mice.

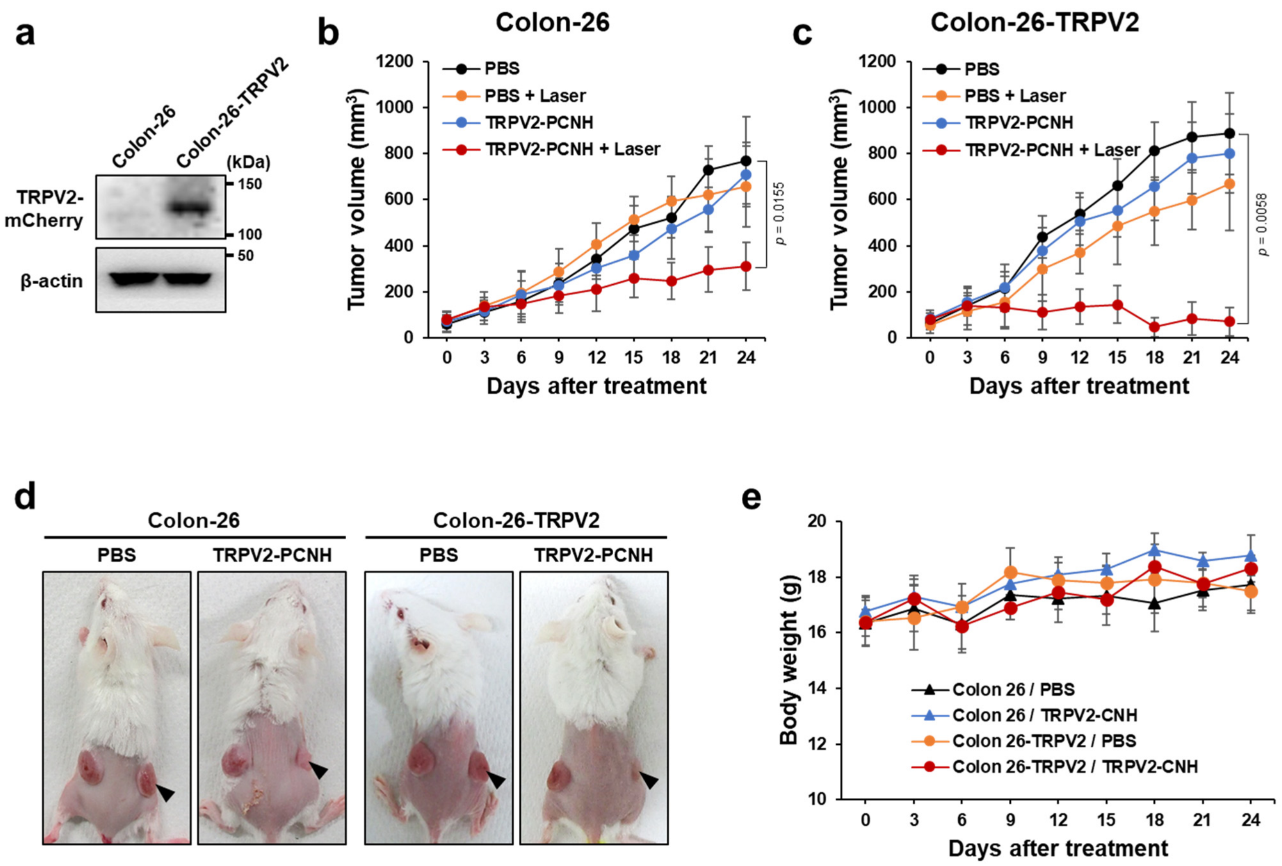

Supplementary Figure 13. (a) Western blotting of TRPV2 in control and TRPV2 transfected cells showing the successful expression of exogenous TRPV2 in Colon-26 cells. (b) Colon-26 and (c) Colon-26-TRPV2 tumor volumes in different groups of mice during treatment (mean ± s.e.m., n = 6 biologically independent mice, two-way ANOVA test). TRPV2-PCNH (5 mg kg^{-1}) was injected (i.p.) every other day, and irradiation was performed with 1064-nm laser at 1 W (~50 mW mm^{-2}) for 5 min at days 2, 5, 10, 16. (d) Photographs of Colon-26 and Colon-26-TRPV2 tumor bearing mice on day 14. Black arrows indicate the tumors irradiated by laser. (e) Average body weight of the mice during the treatment over time. Data are represented as means ± s.e.m.; n = 6 biologically independent mice.

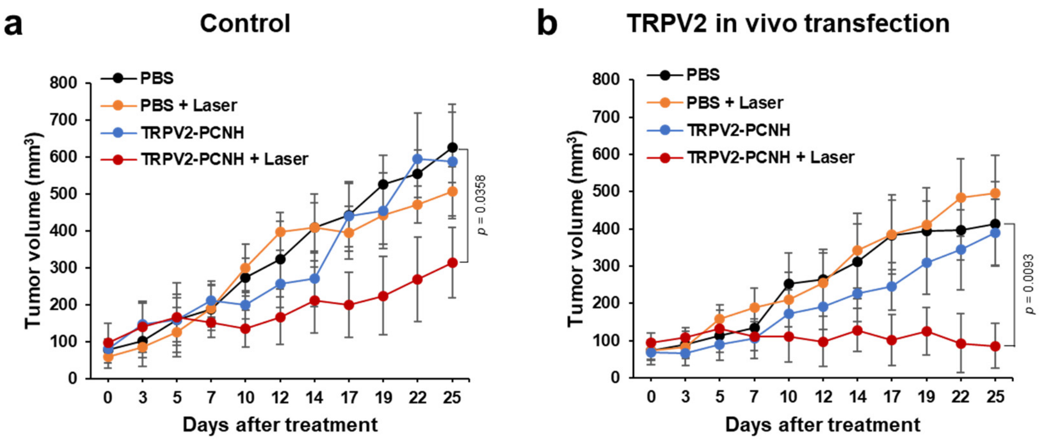

Supplementary Figure 14. Tumour volumes of (a) A549 control and (b) TRPV2 in vivo transfected xenografts in different groups of mice during treatment (mean ± s.e.m., n = 6 biologically independent mice, two-way ANOVA test). TRPV2-PCNH (5 mg kg^{-1}) was injected (i.p.) every other day. In vivo transfection was performed twice a week. Tumours were irradiated with1064-nm laser at 1 W (~50 mW mm^{-2}) for 5 min at days 5, 12, 19.

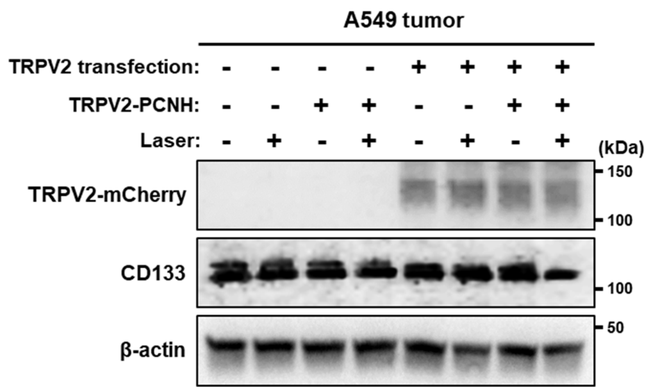

Supplementary Figure 15. Western blotting of CD133 showed its downregulation in A549 tumours that were subjected to in vivo transfection of TRPV2 and nanocomplexes-directed laser irradiation.

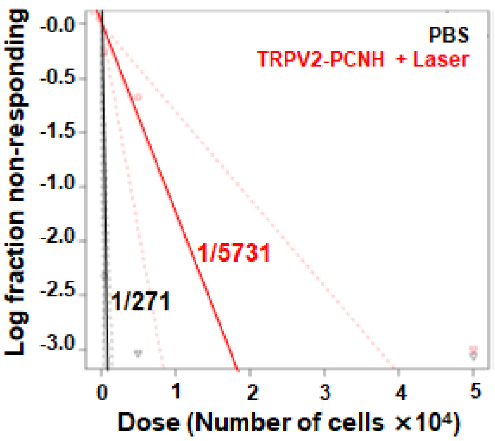

Supplementary Figure 16. Enumeration of tumour-initiating cell frequency by *in vivo* extreme limiting dilution assay. Mice were transplanted with varying doses of HT-29-TRPV2 tumour cells that were dissociated from PBS or TRPV2-PCNH/laser treated xenografts. The estimation was performed at 43 days post-transplantation.

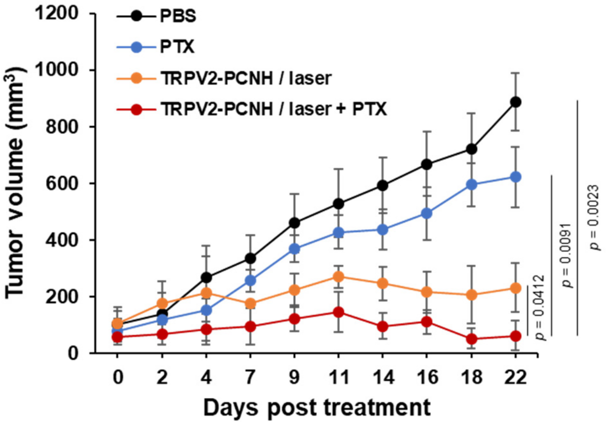

Supplementary Figure 17. Growth curves of TRPV2-A549 xenografts during treatments of PTX (5 mg kg^{-1}), TPRV2-PCNH mediated photo-stimulation (TRPV2-PCNH, 5 mg kg^{-1}; 1064-nm laser, 1 W (~50 mW mm^{-2}) for 5 min at day 3 and 6) and their combination (mean ± s.e.m., n = 5 biologically independent mice, two-way ANOVA test). PBS was served as a negative control.

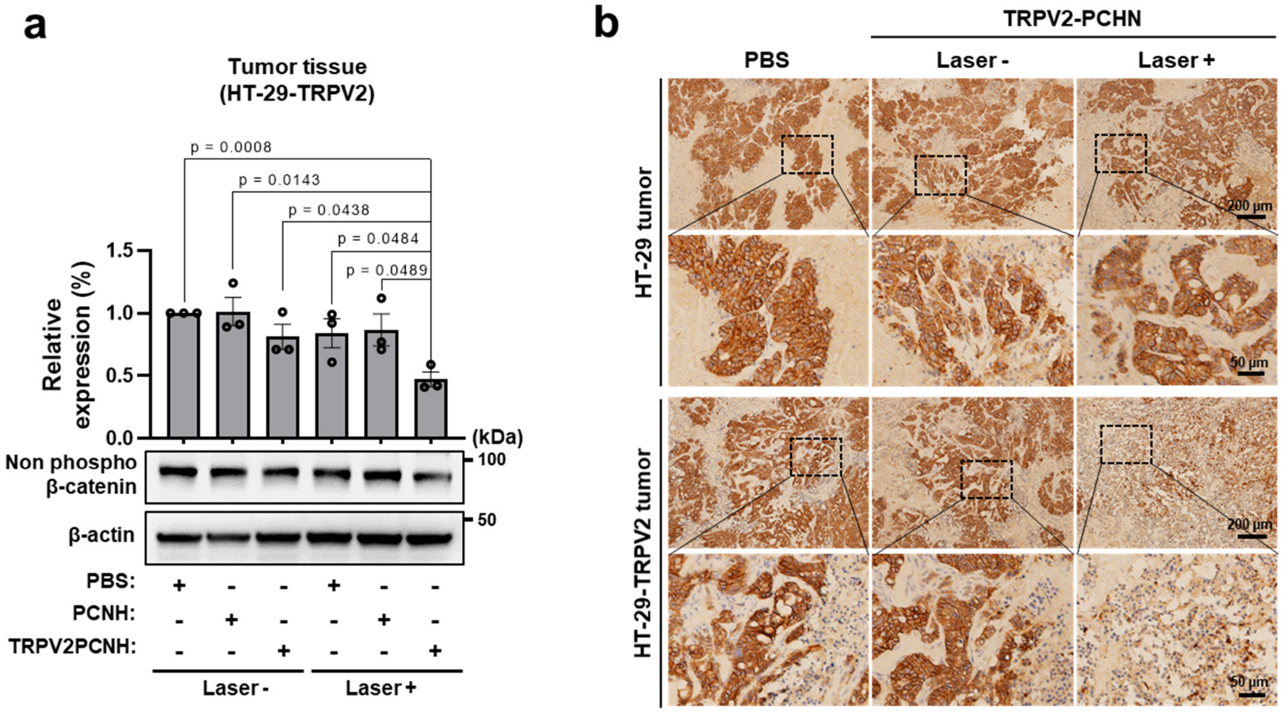

Supplementary Figure 18. (a) Western blotting of β-catenin showed its downregulation in HT-29–TRPV2 tumours treated with nanocomplexes and laser irradiation. Quantitation is shown in the upper panel (mean ± s.e.m., n = 3 independent experiments, Student's two-sided t test). (b) IHC analysis of β-catenin showing its down-regulation in HT-29-TRPV2 xenograft tumour after TRPV2-PCNH-medated NIR treatment.

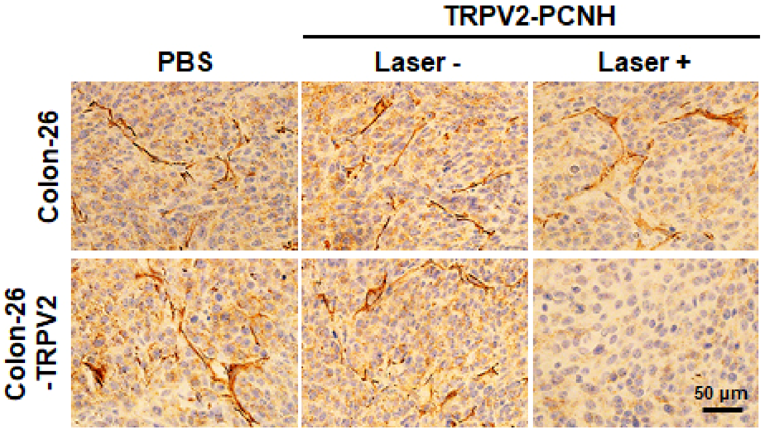

Supplementary Figure 19. IHC staining of β-catenin in Colon-26 control and TRPV2-overexressing tumours showing its down-regulation in the latter after TRPV2-PCNH-medated NIR treatment.

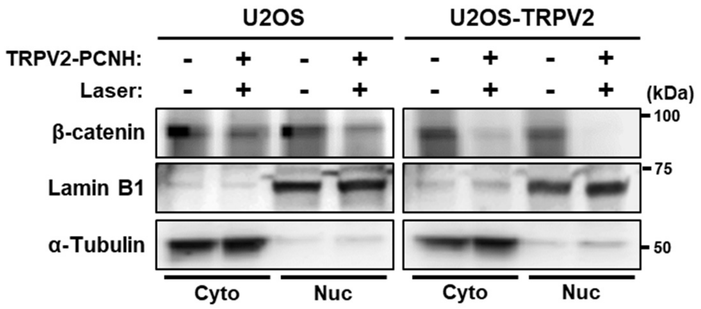

Supplementary Figure 20. Western blotting analysis of cytoplasmic and nuclear levels of β-catenin in U2OS control and TRPV2 transfected cells after treatments of TRPV2-PCNH nanocomplexes and laser irradiation. Lamin B1 and α-Tubulin were used as loading controls for nuclear and cytoplasm protein fractions, respectively.

Supplementary Table 1. Antibodies used in this study

Antibody	Type	Label	Source	Catalog No.	Application
VRL1	Mouse Monoclonal	/	Santa Cruz	sc-390439	Conjugation (20 μg/ml)
TRPV2	Rabbit Polyclonal	FITC	Biorbyt	orb4109	Conjugation (20 μg/ml)
Normal mouse IgG	Mouse Isotype control	/	Sigma-Aldrich	M8642	Conjugation (20 μg/ml)
Cytochrome C	Rabbit Monoclonal	/	Abcam	ab133504	IF (1:500)
VRL1	Rabbit Polyclonal	/	GeneTex	GTX101868	WB (1:1000)
PKCα	Rabbit Monoclonal	/	Abcam	ab32376	WB (1:1000)
β-catenin	Rabbit Monoclonal	/	Cell Signaling	8480	WB (1:1000)<br>IF (1:500)<br>IHC (1:50)
Active β-catenin (Ser33/37/Thr41)	Rabbit Monoclonal	/	Cell Signaling	8814	WB (1:1000)
α-Tubulin	Rabbit Polyclonal	/	Abcam	ab4074	WB (1:2000)
Lamin B1	Rabbit Polyclonal	/	Abcam	ab16048	WB (1:2000)
β-actin	Mouse Monoclonal	HRP	Abcam	ab49900	WB (1:30000)
Anti-Rabbit	Goat Polyclonal	HRP	Santa Cruz	sc-2004	WB (1:5000)
Anti-Rabbit	Goat Polyclonal	Alexa Fluor 488	Thermo Fisher	A-11034	IF (1:500)
Ki-67	Rabbit Monoclonal	/	Thermo Fisher	RM-9106-S	IHC (1:100)
CD133	Rabbit Polyclonal	/	Abcam	ab19898	IHC (1:1200)<br>WB (1:1000)
CD44	Rat Monoclonal	PerCP-Cy5.5	Thermo Fisher	45-0441-82	Flow (1:100)
CD24	Rat Monoclonal	PE	Thermo Fisher	12-0242-82	Flow (1:100)
Rat IgG2b kappa	Rat Isotype control	PerCP-Cy5.5	Thermo Fisher	45-4031-80	Flow (1:100)
Rat IgG2b kappa	Rat Isotype control	PE	Thermo Fisher	12-4031-82	Flow (1:100)

Supplementary Table 2. Primers used in RT-qPCR analyses

Gene	Forward primer (5'→3')	Reverse primer (5'→3')
Nanog	TTTGTGGGCCTGAAGAAAACT	AGGGCTGTCCTGAATAAGCAG
Oct4	CTTGAATCCCGAATGGAAAGGG	GTGTATATCCCAGGGTGATCCTC
CD44	CAGGGACAGCTGCAGCCTCA	ACCTCGTCCCATGGGGTGTG
CD133	GCATTGGCATCTTCTATGGTT	CGCCTTGTCCTTGGTAGTGT
ALDH1	CGCAAGACAGGCTTTTCAG	TGTATAATAGTCGCCCCCTCTC
CD9	ATGATGCTGGTGGGCTTC	GCTCATCCTTGGTTTTCAGC
Axin2	TTATGCTTTGCACTACGTCCCTCCA	CGCAACATGGTCAACCCTCAGAC
C-myc	AATGAAAAGGCCCCCAAGGTAGTTATCC	GTCGTTTCCGCAACAAGTCCTCTTC
Cyclin D1	GAAGATCGTCGCCACCTG	GACCTCCTCCTCGCACTTCT
Survivin	AAGAACTGGCCCTTCTTGGA	CAACCGGACGAATGCTTTT
MMP-7	GAGTGAGCTACAGTGGGAACA	CTATGACGCGGGAGTTTAACAT
Vimentin	CGAGGAGAGCAGGATTTCTC	GGTATCAACCAGAGGGAGTGA
Connexin43	CTACTCAACTGCTGGAGGGAAG	GGCCACCTCAAAGATAGACTTG
Fibronectin	TACACTGGGAACACTTACCG	CCAATCTTGTAGGACTGACC
ZEB1	AAGAATTCACAGTGGAGAGAAGCCA	CGTTTCTTGCAGTTTGGGCATT
18S	CAGGGTTCGATTCCGTAGAG	CCTCCAGTGGATCCTCGTTA

Full scan figure for the western blots

Figure 7b

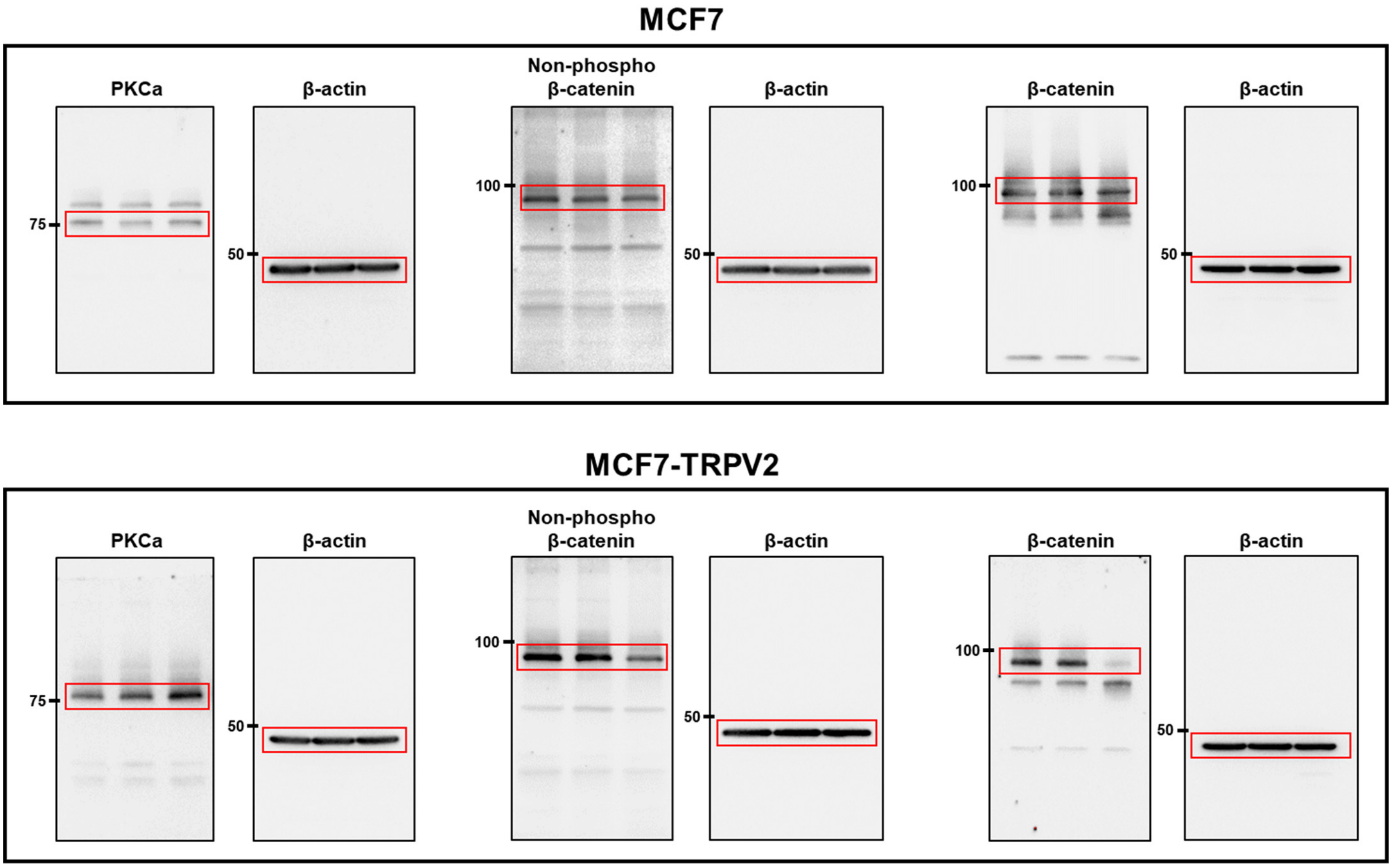

Supplementary figure 2b

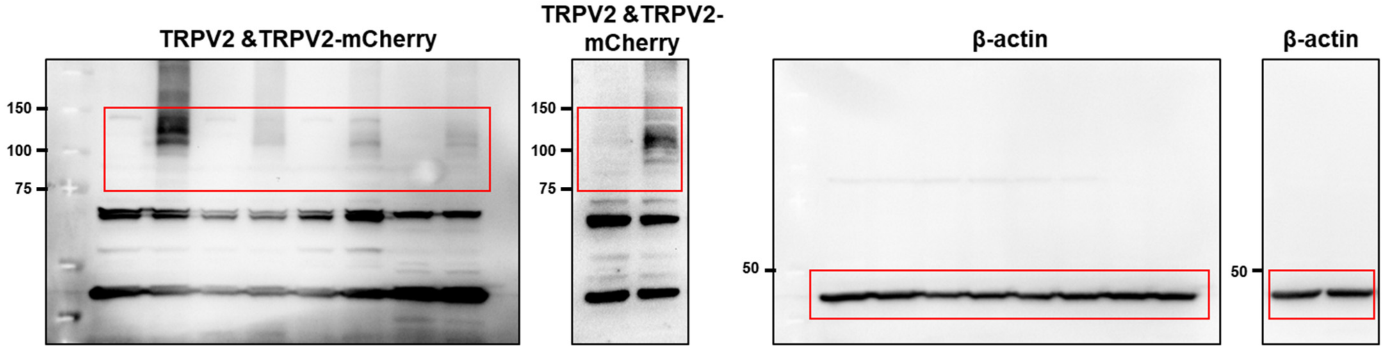

Supplementary figure 13a

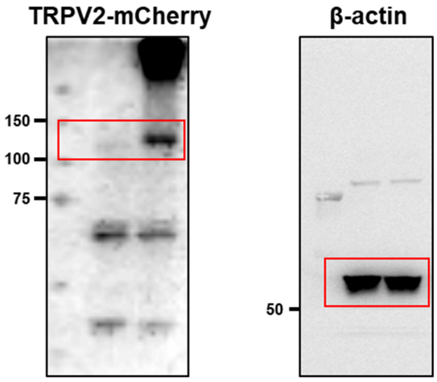

Supplementary figure 15

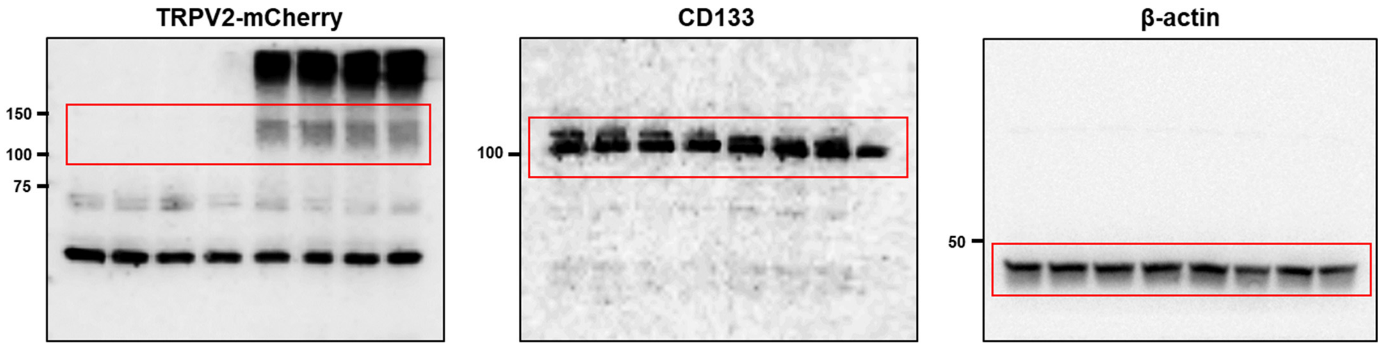

Supplementary figure 18a

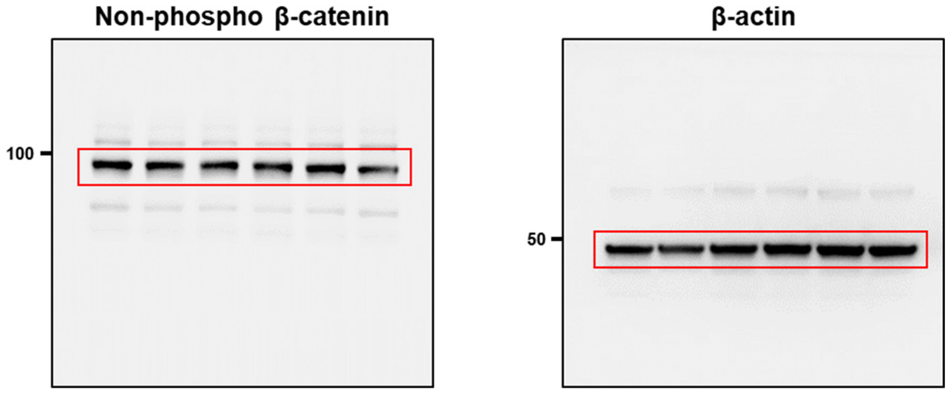

Supplementary figure 20

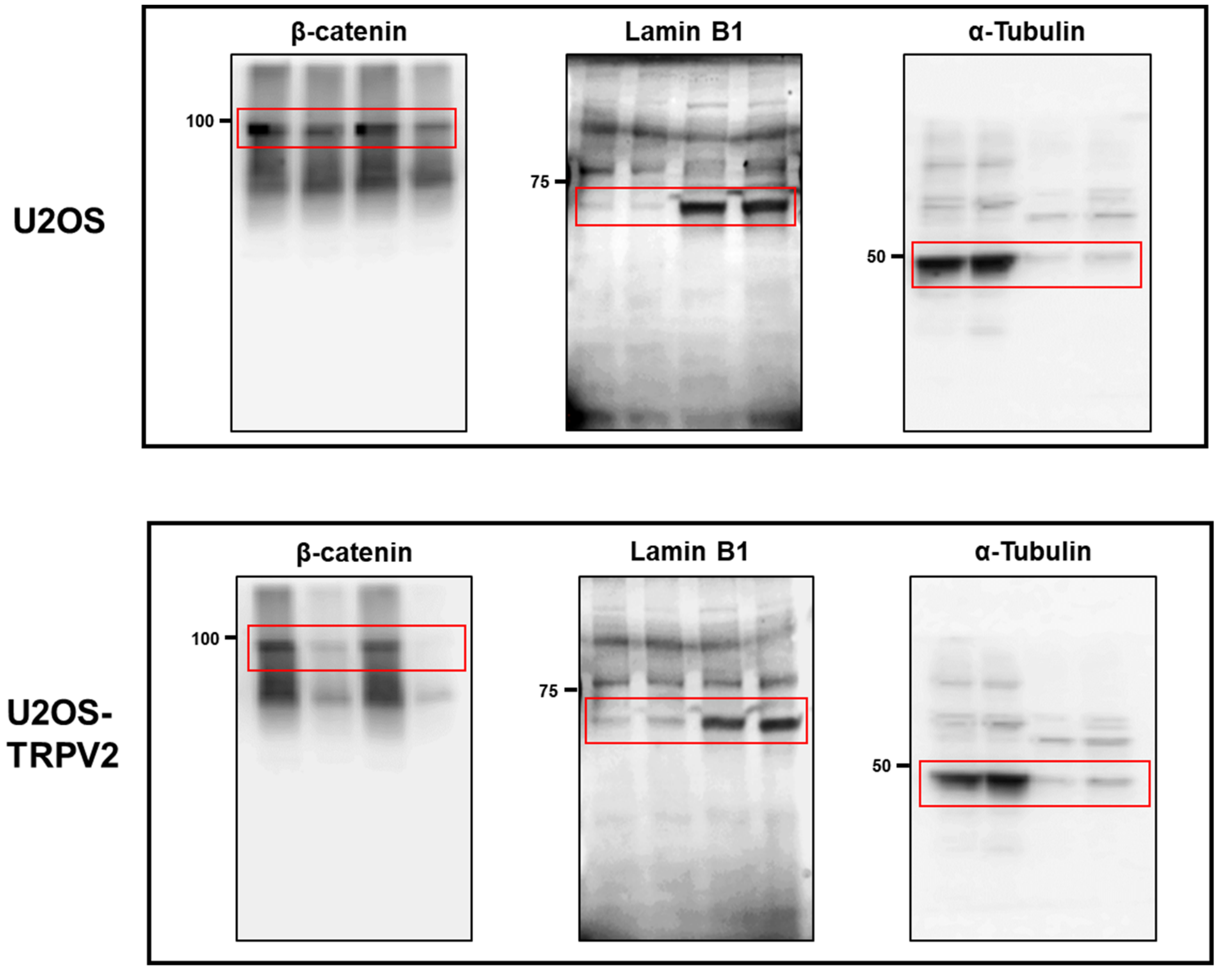

Gating strategy for flow cytometer

Figure 3d and Supplementary figure 6a

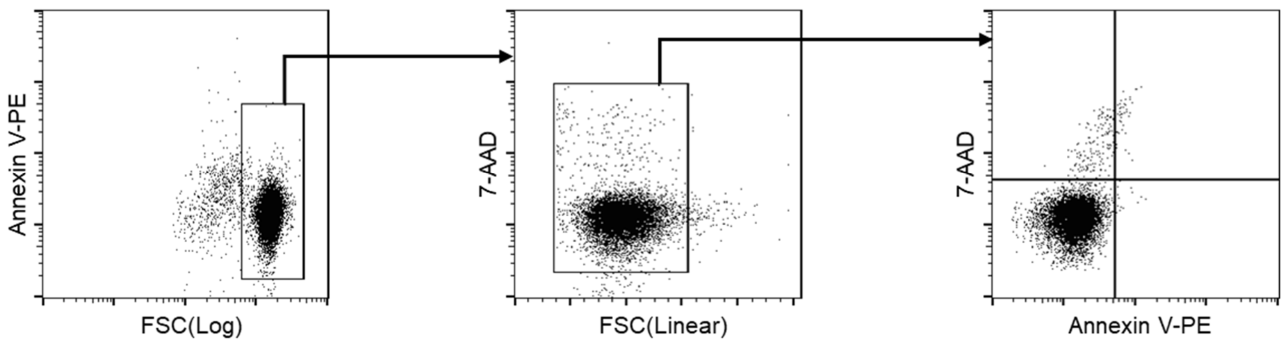

Figure 4f

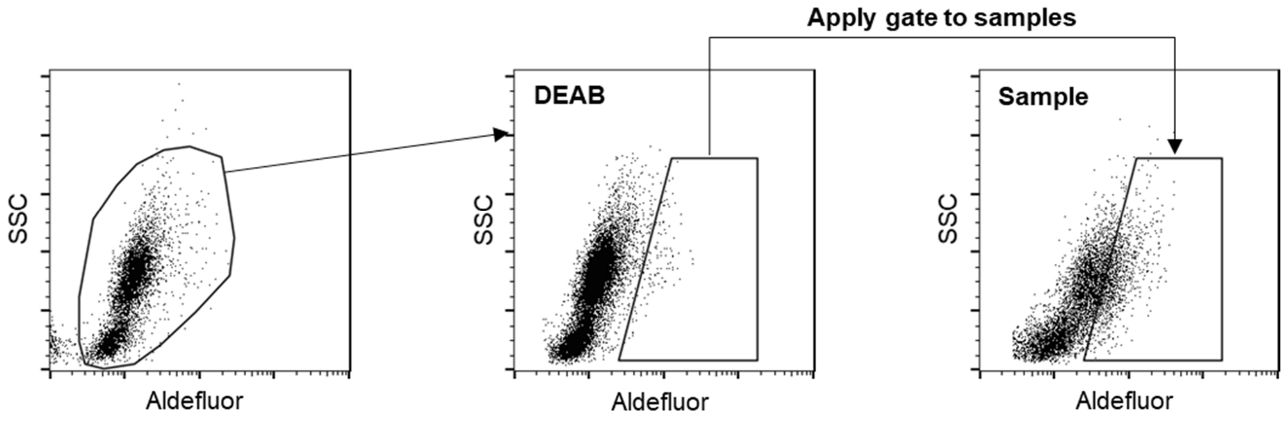

Figure 4g

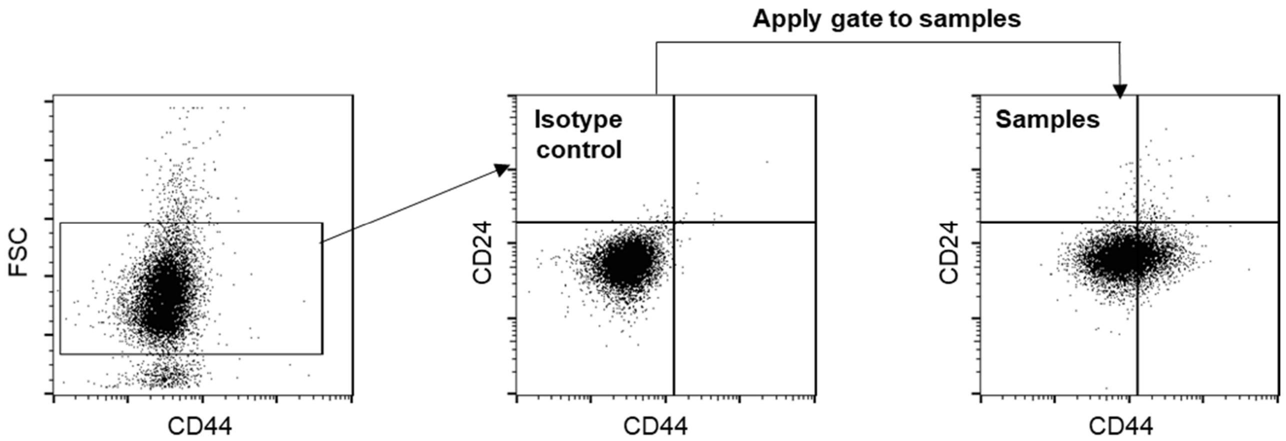

Supplement: Supplementary file 1 — Supplementary Information [file 41467_2020_17768_MOESM1_ESM.pdf]
