# Supplementary material for: Photothermogenetic inhibition of cancer stemness by near-infrared-light-activatable nanocomplexes
Source: Nat Commun. 2020 Aug 17;11:4117. doi: 10.1038/s41467-020-17768-3 (PMC7431860; doi:10.1038/s41467-020-17768-3)
Supplement: Supplementary file 2 — Description of Additional Supplementary Files [file 41467_2020_17768_MOESM2_ESM.docx]

**Supplementary Movie Legends**

File Name: Supplementary Movie 1

Description: Laser-induced U2OS cells

File Name: Supplementary Movie 2

Description: Laser-induced U2OS cells pretreated with TRPV2–PCNH

File Name: Supplementary Movie 3

Description: Laser-induced U2OS–TRPV2 cells

File Name: Supplementary Movie 4

Description: Laser-induced U2OS–TRPV2 cells pretreated with TRPV2–PCNH

File Name: Supplementary Movie 5

Description: Laser-induced C6–TRPV2 cells pretreated with TRPV2–PCNH

File Name: Supplementary Movie 6

Description: Repeated stimulation of U2OS–TRPV2 cells following pretreatments with TRPV2–PCNH
