# Supplementary material for: Photothermogenetic inhibition of cancer stemness by near-infrared-light-activatable nanocomplexes
Source: Nat Commun. 2020 Aug 17;11:4117. doi: 10.1038/s41467-020-17768-3 (PMC7431860; doi:10.1038/s41467-020-17768-3)
Supplement: Supplementary file 9 — Reporting Summary [file 41467_2020_17768_MOESM9_ESM.pdf]

nature research

Corresponding author(s): Eijiro Miyako
Last updated by author(s): Jun 25, 2020

Reporting Summary

Nature Research wishes to improve the reproducibility of the work that we publish. This form provides structure for consistency and transparency in reporting. For further information on Nature Research policies, see our Editorial Policies and the Editorial Policy Checklist.

Statistics

For all statistical analyses, confirm that the following items are present in the figure legend, table legend, main text, or Methods section.

n/a	Confirmed	
☐	☒	The exact sample size (n) for each experimental group/condition, given as a discrete number and unit of measurement
☐	☒	A statement on whether measurements were taken from distinct samples or whether the same sample was measured repeatedly
☐	☒	The statistical test(s) used AND whether they are one- or two-sided<br>*Only common tests should be described solely by name; describe more complex techniques in the Methods section.*
☒	☐	A description of all covariates tested
☒	☐	A description of any assumptions or corrections, such as tests of normality and adjustment for multiple comparisons
☐	☒	A full description of the statistical parameters including central tendency (e.g. means) or other basic estimates (e.g. regression coefficient) AND variation (e.g. standard deviation) or associated estimates of uncertainty (e.g. confidence intervals)
☐	☒	For null hypothesis testing, the test statistic (e.g. F, t, r) with confidence intervals, effect sizes, degrees of freedom and P value noted<br>*Give P values as exact values whenever suitable.*
☒	☐	For Bayesian analysis, information on the choice of priors and Markov chain Monte Carlo settings
☒	☐	For hierarchical and complex designs, identification of the appropriate level for tests and full reporting of outcomes
☒	☐	Estimates of effect sizes (e.g. Cohen's d, Pearson's r), indicating how they were calculated

Our web collection on statistics for biologists contains articles on many of the points above.

Software and code

Policy information about availability of computer code

Data collection	IVIS Living Imaging 3.0; AxioVision SE64 Rel.4.9.1 SP2; Image Lab 6.1; GuavaSoft 3.3; CellSens V3.1; i-control™; Eco Software v5.0
Data analysis	Excel; ChmeDraw 19.1; FLIR Tools; ImageJ; AxioVision SE64 Rel.4.9.1 SP2; GraphPad Prism 8; FlowJo v10.7

For manuscripts utilizing custom algorithms or software that are central to the research but not yet described in published literature, software must be made available to editors and reviewers. We strongly encourage code deposition in a community repository (e.g. GitHub). See the Nature Research guidelines for submitting code & software for further information.

Data

Policy information about availability of data

All manuscripts must include a data availability statement. This statement should provide the following information, where applicable:
- Accession codes, unique identifiers, or web links for publicly available datasets
- A list of figures that have associated raw data
- A description of any restrictions on data availability

The authors declare that all related to this study are available in the article/and or its supplementary information files.

Field-specific reporting

Life sciences study design

All studies must disclose on these points even when the disclosure is negative.

Sample size	No statistical methods were used to predetermine the sample sizes. The number of animals in each group (n = 5-6) was determined according to previous studies cited in our manuscript. The size of each sample is in close agreement with those studies already published and with the need for statistical analysis to discuss the degree of differences and measure the variability of these in vivo data. In previous studies we determined this sample size is sufficient to ensure reproducibility. For all cell based assays, all experiments were performed for more than 3 biological independent samples in triplicate. For blood biochemical, H&E staining, and hematology studies, three mice for each sample were used based on previous studies cited in our manuscript. This size is sufficient because no significant difference was observed between biological replicates.
Data exclusions	No data was excluded from this study
Replication	Experiments were repeated at least three independent experiments with similar results. All experiments were reproduced to reliably support conclusions stated in the manuscript.
Randomization	Cages of mice were randomly selected and then divided into experimental groups for further treatment.
Blinding	Investigators were not blinded to group allocation during experiments.

Reporting for specific materials, systems and methods

We require information from authors about some types of materials, experimental systems and methods used in many studies. Here, indicate whether each material, system or method listed is relevant to your study. If you are not sure if a list item applies to your research, read the appropriate section before selecting a response.

Materials & experimental systems

n/a	Involved in the study	
☐	☒	Antibodies
☐	☒	Eukaryotic cell lines
☒	☐	Palaeontology and archaeology
☐	☒	Animals and other organisms
☒	☐	Human research participants
☒	☐	Clinical data
☒	☐	Dual use research of concern

Methods

n/a	Involved in the study	
☒	☐	ChIP-seq
☐	☒	Flow cytometry
☒	☐	MRI-based neuroimaging

Antibodies

Antibodies used	VRL1; TRPV2; Normal mouse IgG; Cytochrome C; VRL1; PKC-alpha; Beta-catenin; Active beta-catenin (Ser33/37/Thr41); alpha-Tubulin; Lamin B1; beta-actin; Anti-Rabit-HRP; Anti-Rabit-Alexa Fluor 488; Ki-67; CD133; CD44; CD24; Rat IgG2b kappa-PerCP-Cy5.5; Rat IgG2b kappa-PE
Validation	All antibodies were used in the study according to the profile of manufacturers. Antibody validation for preparation of nanoconjugations, immunofluorescence, Immunohistochemistry, flowcytometry or western blotting was validated by the supplier and confirmed in Figure 1, 3, 4, 6, 7, and Supplementary Figures 2, 6, 13, 15, 18, 19, and 20.

Eukaryotic cell lines

Policy information about cell lines

Cell line source(s)	Human bone osteosarcoma (U2OS), lung carcinoma (A549) and normal diploid fibroblasts (TIG3) were obtained from the Japanese Collection of Research Bioresources Cell Bank (Tokyo, Japan). Breast adenocarcinoma (MCF7) and colorectal adenocarcinoma (HT-29) cells were purchased from DS Pharma Biomedical (Tokyo, Japan). Murine colon carcinoma (Colon-26) and rat brain glioma (C6) cells were obtained from the Cell Resource Centre for Biomedical Research, Tohoku University (Sendai, Miyagi, Japan).
Authentication	These cell lines were authenticated by the supplier using STR analysis.
Mycoplasma contamination	No contamination was detected by the supplier using Hoechst DNA stain method, agar culture method, PCR-based assay.
Commonly misidentified lines (See ICLAC register)	These cell lines that we used were not listed in commonly misidentified lines in ICLAC Register.

Animals and other organisms

Policy information about studies involving animals; ARRIVE guidelines recommended for reporting animal research

Laboratory animals	All animal experimental procedures were approved by the Institutional Animal Care and Use Committee of JAIST and AIST. Female BALB/cSlc (4 weeks old), female BALB/cSlc-nu/nu mice (4 weeks old), and female Jcl:ICR mice (6 weeks old) were purchased from Japan SLC (Hamamatsu, Japan).
Wild animals	Irrelevant to experiments.
Field-collected samples	Irrelevant to experiments.
Ethics oversight	No ethical issues.

Note that full information on the approval of the study protocol must also be provided in the manuscript.

Flow Cytometry

Plots

Confirm that:

- [x] The axis labels state the marker and fluorochrome used (e.g. CD4-FITC).
- [x] The axis scales are clearly visible. Include numbers along axes only for bottom left plot of group (a 'group' is an analysis of identical markers).
- [x] All plots are contour plots with outliers or pseudocolor plots.
- [x] A numerical value for number of cells or percentage (with statistics) is provided.

Methodology

Sample preparation	We have described in Methods in more details.
Instrument	Cytomics FC500 flow cytometer (Beckman Coulter) and Guava PCA flow cytometer
Software	FlowJo v10
Cell population abundance	At least 10,000 relevant events were acquired for all FACS analysis.
Gating strategy	In general, cells were first gated on FSC/SSC. Singlet cells were gated using FSC-H and FSC-A. dead cells were then excluded and further surface and intracellular antigen gating was performed on the live cell population.

- [x] Tick this box to confirm that a figure exemplifying the gating strategy is provided in the Supplementary Information.
